# B-box containing protein 1 from *Malus domestica* (MdBBX1) is involved in the abiotic stress response

**DOI:** 10.7717/peerj.12852

**Published:** 2022-02-01

**Authors:** Yaqing Dai, Ying Lu, Zhou Zhou, Xiaoyun Wang, Hongjuan Ge, Qinghua Sun

**Affiliations:** 1 College of Life Science, Shandong Agricultural University, Taian, Shandong, China; 2 Institute of Shandong River Wetlands, Jinan, Shandong, China; 3 Qingdao Academy of Agricultural Science, Qingdao, Shandong, China

**Keywords:** MdBBX1, Malus domestica, Arabidopsis, Abiotic stress, ROS

## Abstract

B-box proteins (BBXs), which act as transcription factors, mainly regulate photomorphogenesis. However, the molecular functions underlying the activity of plant BBXs in response to abiotic stress remain largely unclear. In this investigation, we found that a BBX from *Malus domestica* (*MdBBX1*) was involved in the response to various abiotic stresses. The expression of *MdBBX1* was significantly upregulated in response to abiotic stresses and abscisic acid (ABA). Recombinant MdBBX1 increased stress tolerance in *Escherichia coli* cells. In addition, overexpression of *MdBBX1* in *Arabidopsis* decreased sensitivity to exogenous ABA, resulting in a germination rate and root length that were greater and longer, respectively, than those of wild-type (WT) plants. Moreover, the expression of *ABI5* was decreased in *MdBBX1*-overexpressing lines under ABA treatment. After salt and drought treatments, compared with the WT plants, the *MdBBX1* transgenic plants displayed enhanced tolerance and had a higher survival rate. Furthermore, under salt stress, increased proline (PRO) contents, decreased levels of malondialdehyde (MDA), increased activity of antioxidant enzymes (superoxide dismutase (SOD), peroxidase (POD), catalase (CAT) and ascorbate peroxidase (APX)) and decreased accumulation of reactive oxygen species (ROS) were observed in the *MdBBX1*-overexpressing plants. Overall, our results provide evidence that *MdBBX1* might play a critical role in the regulation of abiotic stress tolerance by reducing the generation of ROS.

## Introduction

B-box containing proteins (BBXs) are typical zinc finger transcription factors with 1 or 2 zinc-binding B-box domain(s) at the N-terminus of protein sequence and occasionally with a CCT domain (for CONSTANS, CONSTANS-like, TOC1) at the C-terminus (Gangappa & Botto, 2014; Khanna et al., 2009). There are 32 family members of BBX in *Arabidopsis thaliana* and they are divided into five subfamilies according to their amino acid sequences. BBXs in group I-III contain a CCT domain that participates in the regulation of the transcription or nuclear import. Groups I, II, and IV contain two B-box motifs, while groups III and V harbor only one B-box motif (Gangappa & Botto, 2014; Khanna et al., 2009). BBX members in different groups have been identified to function in regulating anthocyanin accumulation, flowering, shade avoidance and photomorphogenesis, as well as responses to stress (An et al., 2019; An et al., 2020; Chang et al., 2008; Crocco et al., 2011; Crocco et al., 2015; Datta et al., 2007; Datta et al., 2008; Fang et al., 2019; Gangappa et al., 2013; Heng et al., 2019a; Heng et al., 2019b; Sarmiento, 2013; Wang et al., 2013; Wei et al., 2016; Yadav et al., 2019; Zhang et al., 2017).

In the BBX family of *Arabidopsis*, the members in the same group may have different functions. For example, both BBX21 and BBX24 belong to the same structural group, group IV (whose members have two B-box motifs and no CCT domain), but their functions in regulating photomorphogenesis are opposite. BBX21 is a positive regulator of photomorphogenesis, whereas BBX24 is a negative regulator (Xu et al., 2016). Despite the opposite functions of BBX21 and BBX24, the antagonistic modulating ability of both depends on HY5 (Job et al., 2018), which is a central downstream regulator of light-mediated developmental processes and can bind to the promoter of the *ABI5* gene to activate its expression (Chen et al., 2008). To illustrate the molecular mechanism for underlying the contrasting functions of BBX21 and BBX24 (Job et al., 2018), the protein sequences of these two genes were compared, and the results revealed that their functional differences were mainly determined by different sequences of the C-terminal region. In support of this notion, the researchers constructed two vectors that expressed BBX24 and BBX21 proteins fused to each other’s C-terminal sequences; the fusion proteins were subsequently named “BB24C21” and “BB21C24”, respectively. The results showed that, similar to the overexpression of BBX21, the overexpression of BB24C21 could transcriptionally upregulate the expression of HY5, whereas over-expression of BB21C24 did not have any effect on the mRNA levels of *HY5* (Job et al., 2018). Furthermore, the researchers found that BBX21 could mediate HY5 post transcriptionally. In contrast, BBX24 could prevent HY5 from binding to the promoter of the target gene, probably by heterodimerizing with HY5 and inhibiting its ability to bind to DNA. In conclusion, closely related BBXs may perform opposite functions.

BBXs are also involved in the stress response (Gangappa & Botto, 2014). Act as a kind of salt tolerance-related protein, BBX24 can negatively regulate the expression of many stress-related genes (Nagaoka & Takano, 2003). *AtBBX24* transgenic plants were shown to be more salt tolerant than wild-type (WT) plants under salt stress. BBX5, a group I member, contains two B-box domains and one CCT domain, and is involved in the response to abiotic stress through the abscisic acid (ABA)-dependent signaling pathway. Overexpressing *BBX5* can notably improve the plant resistance to abiotic stresses (Min et al., 2015). Overexpression of a BBX protein in banana obviously improved its tolerance to biotic and abiotic stresses, such as pathogen infection and chilling (Chen et al., 2012). Similarly, overexpression of *OsBBX25* in *Arabidopsis thaliana* can increase plant tolerance to abiotic stresses (Liu et al., 2012). Heterologous expression of *AtBBX21* enhances the photosynthesis rate and alleviates photoinhibition in *Solanum tuberosum* (Crocco et al., 2018). In addition, some tomato *BBX* genes can also be induced in response to heat, drought and phytohormones (Chu et al., 2016). Overall, BBX proteins play vital roles in regulating various stress responses.

Our previous study reported that there are 64 *BBXs* in the apple genome, which can be divided into five groups, similar to the *Arabidopsis* BBX family. Some *MdBBX* genes are induced in response to different abiotic stresses, indicating that *MdBBXs* may participate in abiotic stress responses (Liu et al., 2018). A recent study found that *MdBBX10* from apple could promote tolerance to drought and salt stresses in *Arabidopsis* (Liu et al., 2019a). MdBBX10 belongs to group V, and contains one B-box domain, but no CCT domain. Overexpression of *MdBBX10* in *Arabidopsis* dramatically improved the tolerance to abiotic stress and increased sensitivity to ABA during the seed germination and seedling stages (Liu et al., 2019a). Here, we demonstrated that a BBX member of group I, MdBBX1, which contains two B-box domains and a CCT domain (Fig. S1), also responds to abiotic stress, but causes insensitivity to exogenous ABA when overexpression in *Arabidopsis*, which is opposite to the response of MdBBX10 to ABA.

## Materials and Methods

### Plant growth conditions and treatments

For organ-specific expression analyses, different apple organs, including roots, stems, leaves, flowers and fruits, were sampled from 6-year-old apple trees growing at the experimental station of Shandong Agricultural University (Tai’an, Shandong, China).

Apple (*golden delicious*) seedlings were cultivated under greenhouse conditions (relative humidity of 60–75%) at 22 ± 1 °C with a 16 h light/8 h dark photoperiod for approximately 1 year. Then, the uniformly growing seedlings were selected for stress treatments. For salt and drought stress treatments, the apple seedlings were watered with solutions of 250 mM NaCl or 25% (w/v) polyethylene glycol-6000 (PEG-6000), and control seedling received the same amount of water only. For ABA treatment, 100 µM ABA solutions were directly sprayed on the seedlings. For cold stress, the apple seedlings were subjected to 4 °C condition, while seedlings growing at room temperature (25 °C) were used as the controls. Samples were collected from three different kinds of seedlings at 0, 3, 6, 9 and 12 h after treatment, as was done in a previous study (Yuan et al., 2013). Then, the collected samples were immediately frozen in liquid nitrogen and stored at −70 °C till used. Subsequently, the total RNA was extracted from the collected samples using an improved cetyl-trimethylammonium bromide (CTAB) procedure (Gasic, Hernandez & Korban, 2004).

The seeds of WT (Col-0) and transgenic *Arabidopsis* were disinfected and sown on 1/2 Murashige and Skoog (MS) media. After culturing at 4 °C for 3 days to undergo vernalization, the seedlings were transferred to a greenhouse condition, which included a 22 ± 1 °C temperature with a 16 h light/8 h photoperiod. Then, 3-week-old WT and transgenic plants were treated with 250 mM NaCl or 25% (w/v) PEG-6000 as described by Liu et al. (2019a), and the control seedlings were treated with water only. Plant growth status was observed and the survival rates were determined daily. Each treatment was performed at least three times.

### Quantitative real-time PCR (qRT-PCR) analysis

QRT-PCR is a commonly used approach for the quantitative detection of gene expression in real time (Deepak et al., 2007). All the primers used in this investigation were designed according to the target gene sequences *via* the Beacon Designers software and were shown in Table S1. qRT-PCR was carried out using a SYBR® PrimeScript™ RT-PCR Kit (TaKaRa, Dalian, China) and run on a CFX96TM Real-Time PCR Detection System (Bio-Rad, Hercules, CA, USA). The *Arabidopsis* *Actin8* and apple *actin* genes were used as reference genes (Wang et al., 2016). The thermal cycling parameters were as follows: 40 cycles of 95 °C denaturation for 15 s, 55 °C annealing for 20 s and 70 °C extension for 15 s. The qRT-PCR data were analyzed by the 2^−ΔΔCt^ method (Livak & Schmittgen, 2001). The relative expression of *MdBBX1* in the treated samples was compared with that in the nontreated samples at each treatment time point with significant differences (*P* < 0.05) determined based on Tukey’s multiple test.

### Subcellular localization of *MdBBX1*

The full-length coding sequence of *MdBBX1* was amplified from apple and inserted into the pROKII vector containing a GFP gene and the *CaMV35S* promoter. Cells of *Agrobacterium tumefaciens* GV3101 with the recombinant plasmid were cultured overnight, resuspended in osmotic solution (10 mM, Mole MgCl_2_, 10 mM 2-[N-morpholino] ethanesulfonic acid (MES) and 150 mM acetosyringone), and then injected into the leaves of 1-month-old *Nicotiana benthamiana* plants. The fluorescent signal of *MdBBX1*-GFP was detected *via* a confocal microscope (LSM 510 META, Carl Zeiss, Jena, Germany) after 2–3 days. The nuclei were subsequently stained with 100 g/mL 4′, 6 -diamidino-2-phenylindole (DAPI) (Solarbio, Beijing, China) for 10 min. Leaves overexpressing 35S-GFP were used as controls (Wang et al., 2018).

### Construction of expression plasmids

The cDNA sequence of *MdBBX1* was inserted into the polyclonal sites of pET-30a (+) (Novagen), which contained His-tagged sequences. Then, the recombinant vector was transformed into *Escherichia coli* BL21 cells. The recombinant sequences in the plasmids were sequenced by Sangon Biotechnology Company (Shanghai, China).

### Survival test of *Escherichia* under different abiotic stresses

Survival analysis of *Escherichia coli* under salt and drought stress was conducted as described by Du et al. (2014). The cells were cultured in Luria-Bertani (LB) liquid media until the OD_600_ reached 0.4–0.6, and then the expression of the recombinant protein was induced for 2 h using isopropyl β-D-1-thiogalactopyranoside (IPTG) at 37 °C. All the bacterial cultures were first diluted to an OD_600_ of 0.6 and then diluted 10^−3^, 10^−4^ and 10^−5^ times. For the survival test on solid media, 10 µL cultures of each dilution were spotted onto solid LB media that included 500 mM KCl, 500 mM NaCl or 600 mM mannitol and incubated for 12 h at 37 °C. Then, the colony numbers in each dish for the culture diluted to 10^−5^ were counted. Each experiment was performed at least three times.

For the survival test in liquid media, the cultures were first diluted to an OD_600_ of 0.6, after which 200 µL of the cultures were put into 20 mL of LB solution that included 500 mM NaCl, 500 mM KCl or 600 mM mannitol and incubated at 37 °C on a rotary shaker (150 rpm). Then, the bacterial suspension was collected every 2 h for 24 h, after which the OD_600_ of the culture was measured. Each experiment was repeated at least 3 times.

### Generation of transgenic plants

The pROKII-*Md*BBX1 recombinant plasmids were transformed into *Arabidopsis* in accordance with the floral-dip method *via Agrobacterium tumefaciens* (GV3101)-mediated transformation. Subsequently, the *MdBBX1* overexpression seedlings were screened on MS agar media supplemented with 50 µg/mL kanamycin and were further identified *via* PCR using *MdBBX1* and GFP primers. The specific primers used are shown in the Table S1.

### Analysis of germination status under different abiotic stresses

Fifty seeds of WT or overexpression (OE) lines were sown onto 1/2-strength MS agar media supplemented with different concentrations of mannitol (300 or 400 mM), NaCl (150 or 200 mM) or ABA (0.2 or 0.6 µM). Seed germination was observed and measured every 12 h. For root length analysis, the seeds were grown vertically on 1/2-strength MS media as described above. The root length of 20 seedlings was measured after 10 days, and each treatment was performed at least three times.

### Measurements of proline (PRO), malondialdehyde (MDA), and reactive oxygen species (ROS) contents and antioxidant enzyme activity

For physiological index measurements, the free PRO content was measured using a spectrophotometric PRO kit (Solarbio Life Sciences, Beijing, China). The MDA content was measured using a thiobarbituric acid reactive substances assay (Aguilar Díaz de León & Borges, 2020; Hodges, Delong & Prange, 1999) and the contents of hydrogen peroxide (H_2_O_2_) and superoxide anions (O_2_^.−^) were measured using O_2_^.−^ and H_2_O_2_ kits, respectively (Nanjing Jiancheng Bioengineering Institute, China). Similarly, the total protein contents were determined using a BCA Protein Assay Kit (Nanjing Jiancheng Bioengineering Institute, Nanjing, China), and the activities of superoxide dismutase (SOD), peroxidase (POD), catalase (CAT) and ascorbate peroxidase (APX) were measured based on the protocols of the corresponding kits (Nanjing Jiancheng Bioengineering Institute, Nanjing, China) (Bai et al., 2020; Li et al., 2019; Ma et al., 2019).

### Statistical analysis

All experiments were conducted at least three times. The data presented are the means ± standard deviations of three replications. Statistical significance was analyzed using SPSS software (version 17.0), and Turkey’s multiple range comparison tests were performed to determine the significance of differences between samples (*P* < 0.05 or *P* < 0.01).

## Results

### Organ-specific expression pattern analysis and subcellular localization of MdBBX1

**Figure 1:**
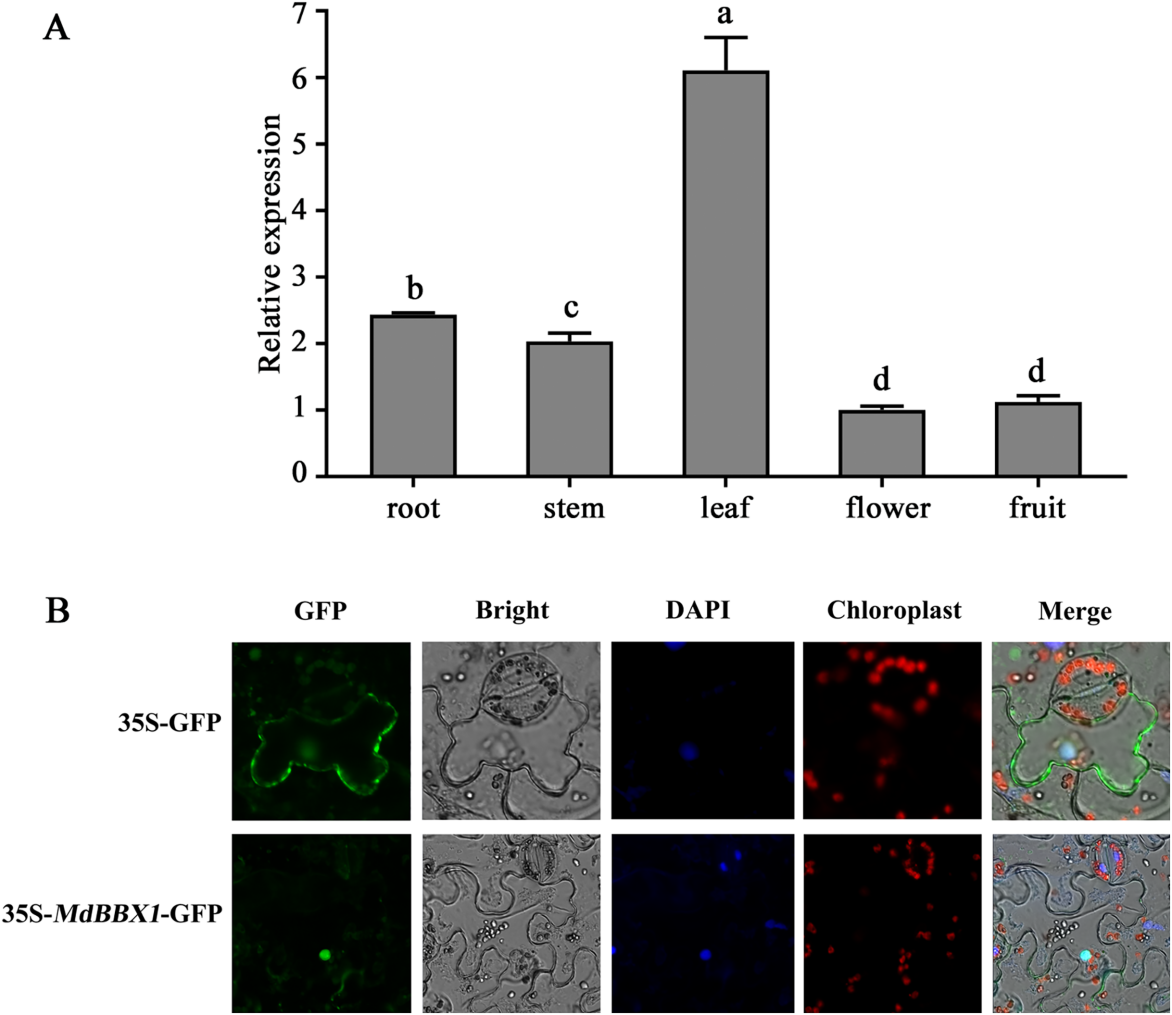
Expressional pattern and subcellular localization analysis of MdBBX1. (A) qRT-PCR analysis of *MdBBX1* expression in different organs of 6-year old apple seedlings. The experiments were repeated three times and vertical bars indicate the standard error of the mean. The letters above the columns represent significant differences (*P* < 0.05) based on Tukey’s multiple test. (B) Subcellular localization of MdBBX1-GFP fusion protein. *35S::MdBBX1-GFP* construct was transformed into tobacco leaves and was examined in the epidermal cells at 48 h after the transformation by confocal fluorescence microscopy. The nuclei were stained with 100 g/mL DAPI for 10 min.

The organ-specific expression pattern of *MdBBX1* was analyzed *via* qRT-PCR. The results revealed that the transcript level of *MdBBX1* was obviously higher in the leaves than in other organs (Fig. 1A). To identify the subcellular localization of MdBBX1, an MdBBX1-GFP construct and empty GFP plasmid were introduced into epidermal cells of tobacco leaves, after which the nuclei were stained by DAPI. The fluorescence signal and DAPI staining were predominantly distributed in the nucleus (Fig. 1B), which indicated that MdBBX1 was localized there.

### The *MdBBX1* promoter contains elements related to the abiotic stress response

Some *BBX* members were found to respond positively to abiotic stresses in a previous study (An et al., 2020; Crocco & Botto, 2013; Liu et al., 2018; Shalmani et al., 2018). To explore the potential functions of *MdBBX1* in response to a variety of abiotic stresses, the DNA sequence within 2000 bp upstream of *MdBBX1* (the promoter sequence) was scanned *via* PlantCARE software (http://bioinformatics.psb.ugent.be/webtools/plantcare/html/). The results showed that many *cis*-acting elements that may be involved in responses to abiotic stress, light and other signals were present in the *MdBBX1* promoter region (Table 1). For instance, MBS (MYB-binding site) elements are involved in the response to drought stress, and ABA-responsive elements (ABREs) function in response to exogenous ABA. LTRs are found to participate in low-temperature responsiveness. In addition, some *cis*-acting elements such as TC-rich repeats and TCA elements are involved in responses to defense and stress or to salicylic acid. Taken together, these results indicated that *MdBBX1* may participate in the response to abiotic stress.

**Table 1:** Putative *cis*-acting elements of the promoter of *MdBBX1*.

*Cis*-element	Position	Sequence(5′-3′)	Function
ABRE	−1331	ACGTG	*cis*-acting element involved in the abscisic acid responsiveness
CGTCA-motif	−1794	CGTCA	*cis*-acting regulatory element involved in the MeJA-responsiveness
G-Box	−1330	CACGTT	*cis*-acting regulatory element involved in light responsiveness
GT1-motif	−1523	GGTTAAT	light responsive element
LTR	−1992	CCGAAA	*cis*-acting element involved in low-temperature responsiveness
MBS	−577	CAACTG	MYB binding site involved in drought-inducibility
P-box	−1778	CCTTTTG	gibberellin-responsive element
TC-rich repeats	−941	ATTCTCTAAC	*cis*-acting element involved in defense and stress responsiveness
TCA-element	−1242	CCATCTTTTT	*cis*-acting element involved in salicylic acid responsiveness

**Figure 2:**
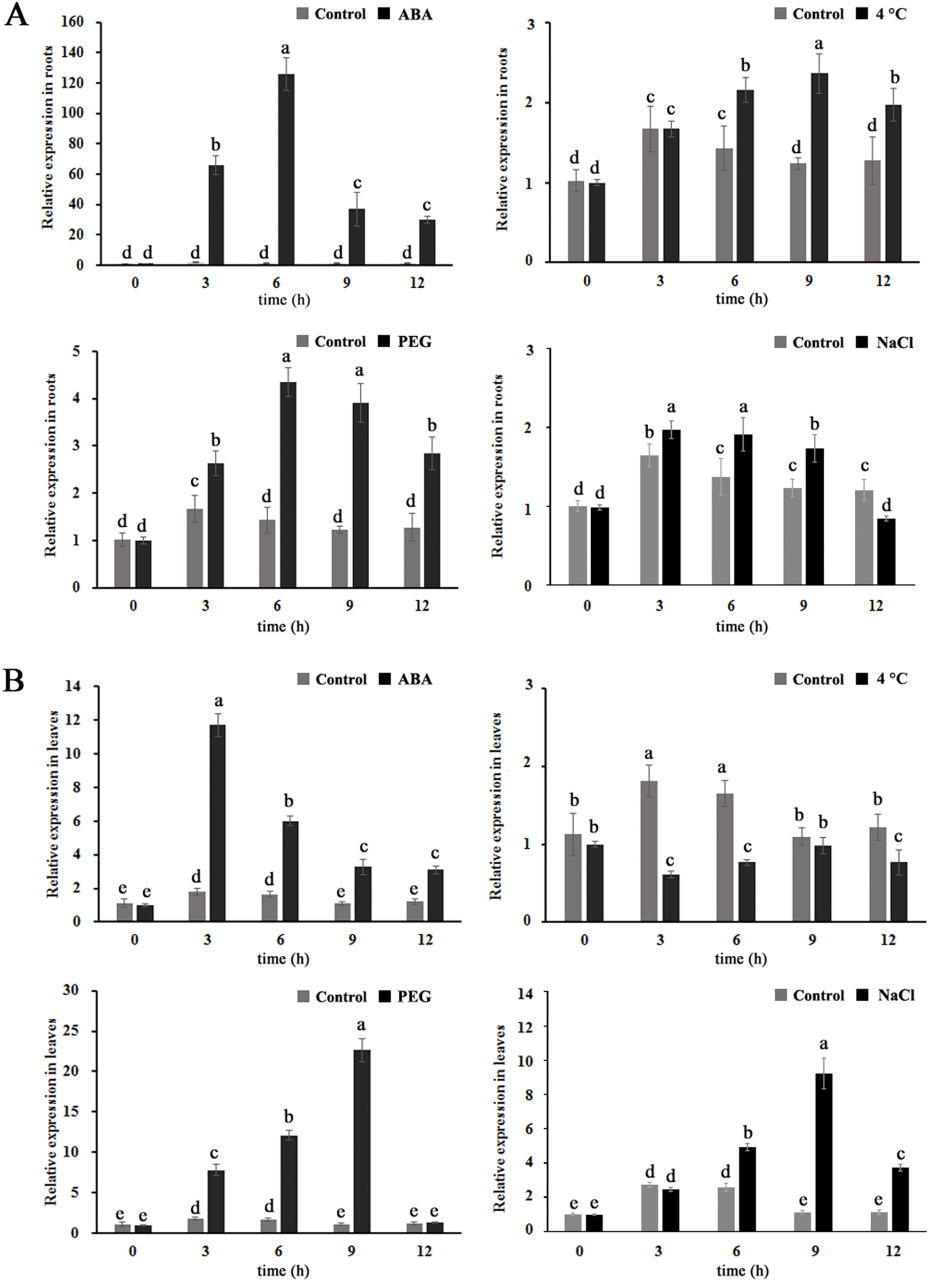
The expression pattern of *MdBBX1* in (A) roots and (B) leaves under various abiotic stresses. Each column represents the mean values of three biological replicates and vertical bars indicate the standard error of the mean. The letters above the columns represent significant differences (*P* < 0.05) based on Tukey’s multiple test.

To further investigate whether *MdBBX1* is expressed in response to abiotic stress, apple seedlings were treated with solutions of 100 µM ABA, 25% polyethylene glycol (PEG), or 250 mM NaCl or 4 °C for different durations. As shown in Fig. 2, the transcript levels of *MdBBX1* were upregulated in both the leaves and the roots under exogenous ABA, salt and PEG treatment, *MdBBX1* expression was maximized after 6 h of stimulation by ABA, PEG or NaCl compared with the control in the roots and increased by approximately 120-, 4- and 2-fold, respectively. In addition, the transcript levels of *MdBBX1* were upregulated by 12-, 20- and 10-fold after treatment with ABA, PEG or NaCl in the leaves. Notably, the expression of *MdBBX1* was significantly upregulated under cold conditions only in the roots. Taken together, the above results showed that the expression of *MdBBX1* was induced in response to different abiotic stresses, which suggested that *MdBBX1* may be involved in the response to abiotic stress.

### Ectopic expression of *MdBBX1* in *Escherichia* improved cell tolerance to abiotic stress

**Figure 3:**
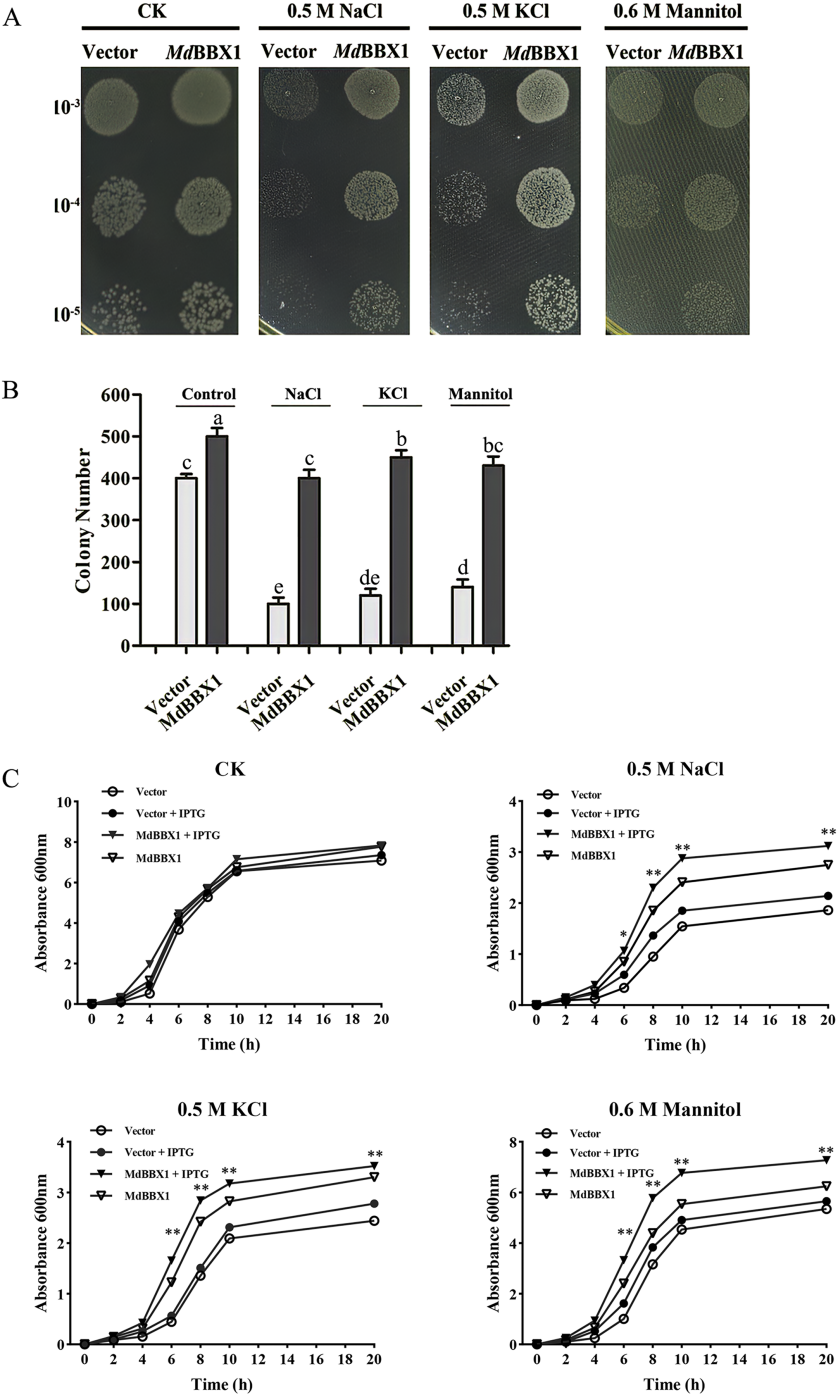
Survival test of *E. coli* cells carrying *MdBBX1* or empty vector under various stress conditions. (A) A total of 10 μL cultures induced by 1 mM IPTG for 2 h (OD600 about 0.5) were diluted from 10^−3^ to 10^−5^ and were spotted on solid medium containing NaCl, KCl or mannitol. Each experiment was carried out in three biological replicates. (B) The colony numbers appearing on above medium were counted in 10^−5^ concentration. Mean values are from three independent replicates and error bars indicate standard deviation. The letters above the columns represent significant differences (*P* < 0.05) based on Tukey’s multiple test. (C) Growth curve of *E. coli* cells in LB liquid medium with and without addition of 1 mM IPTG under NaCl, KCl or mannitol treatments. The mean expression value was calculated from three independent replicates. Vertical bars indicate the standard error of mean, ** and * indicate significant differences compared with vector cells at *P* < 0.01 and *P* < 0.05, respectively.

To determine the stress resistance function of *MdBBX1*, heterogeneous expression of *MdBBX1* was induced in *Escherichia coli* growing on solid media under different stress conditions. Survival tests of *Escherichia coli* cells were carried out, with empty vectors used as controls. As shown in Figs. 3A–3B, the growth of the cells in nonstress media showed slight significant difference between those harboring *MdBBX1* and those harboring the empty vector. However, when the same concentration of cultures was inoculated onto plates with stress media, the number of *Escherichia coli* colonies expressing *MdBBX1* was significantly higher than that of the control colonies harboring the empty vector. To further confirm the function of *MdBBX1*, a growth curve of *Escherichia coli* in liquid media was constructed. As shown in Fig. 3C, under nonstress conditions, few differences were observed among the growth curves of cells with and without *MdBBX1* expression. However, under different stress conditions, the growth rate of *Escherichia coli* expressing *MdBBX1* was significantly faster than that of the control cells carrying the empty vector. The results suggested that *MdBBX1* provided strong abiotic stress tolerance.

### Overexpression of *MdBBX1* increased resistance to abiotic stress in *Arabidopsis*

**Figure 4:**
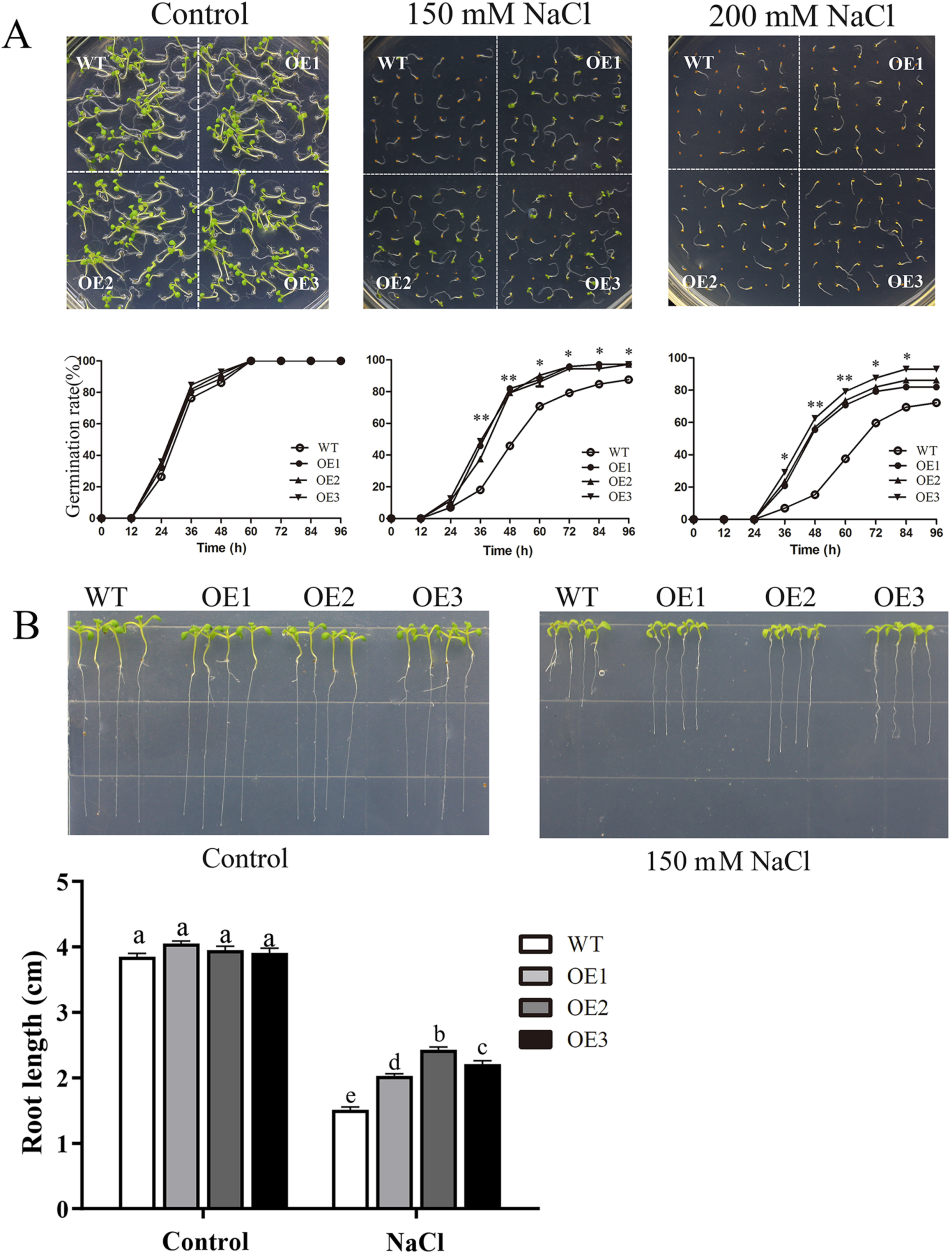
Germination and root length phenotypes of *MdBBX1* overexpression plants under salt tolerance. (A) Germination phenotype of the WT and *MdBBX1* -overexpressed (OE) lines on 1/2 MS medium containing NaCl (0, 150 and 200 mM). Three independent experiments were conducted and each phenotype included 50 seeds. The mean expression value was calculated from three independent replicates. Vertical bars indicate the standard error of mean, **P < 0.01 and *P < 0.05 compasred with WT. (B) The root length of WT and *MdBBX1*-transgenic lines in 1/2 MS medium containing 150 mM NaCl. Root growth was measured after NaCl treatment for 14 days. The letters above the columns represent significant differences (*P* < 0.05) based on Tukey’s multiple test.

**Figure 5:**
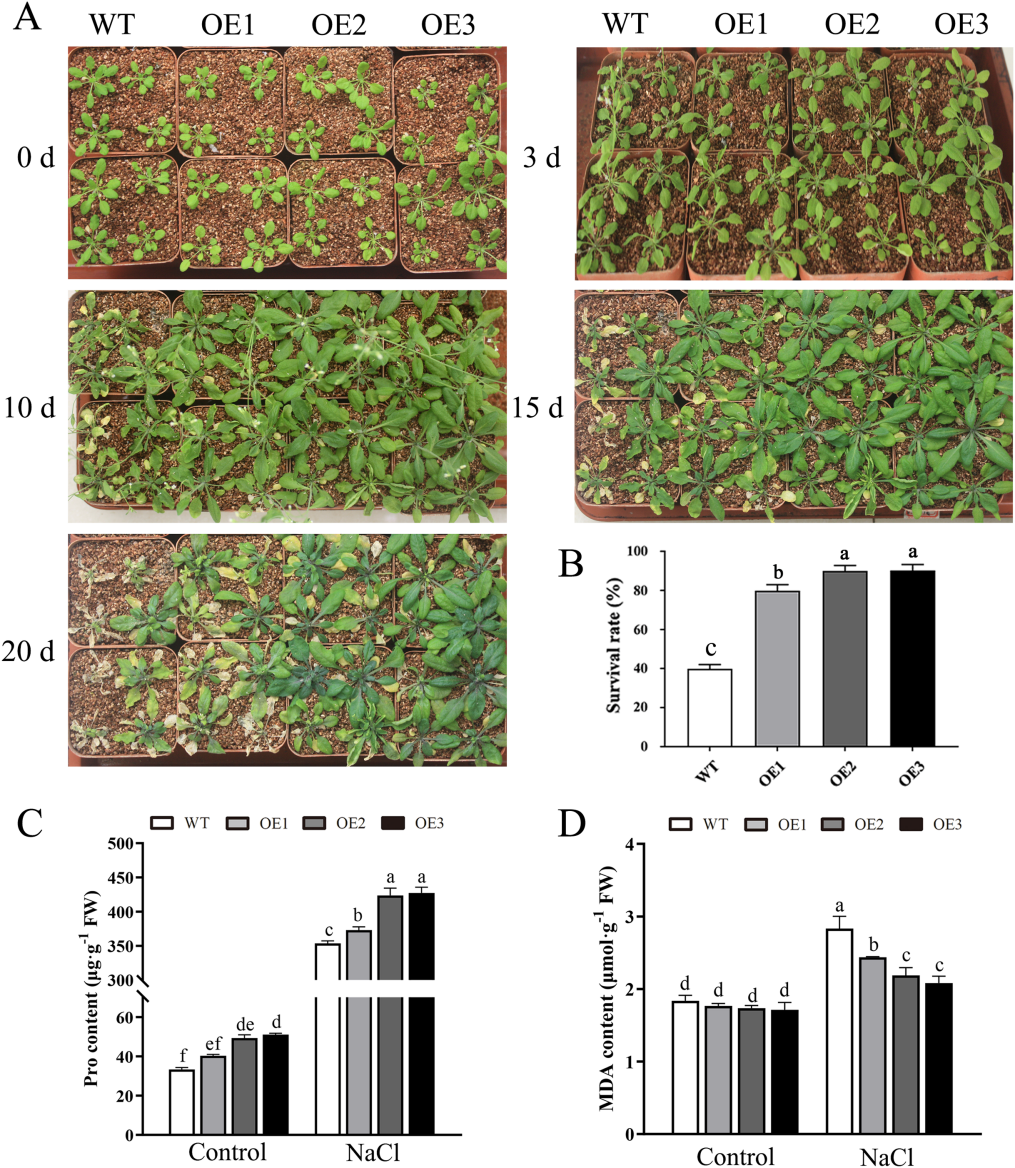
Overexpression *MdBBX1* enhanced salt tolerance in transgenic plants. (A) The representative phenotypes of 3-week old WT and OE seedlings were treated with NaCl (250 mM) for 3 days to 20 days. (B) Survival rates of WT and transgenic plants after salt stress. PRO (C) and MDA content (D) were measured in WT and transgenic plants after salt stress. Mean values are from three independent replicates and error bars indicate standard deviation. The letters above the columns represent significant differences (*P* < 0.05) based on Tukey’s multiple test.

To determine the role of *MdBBX1* in abiotic stress resistance in plants, three transgenic lines (OE1, OE2 and OE3) with similar expression levels of *MdBBX1* were obtained and subjected to salt and drought treatment (Fig. S1). As shown in Fig. 4A, on normal media, the germination rate and growth status of seedlings exhibited no obvious differences between the WT and transgenic lines. However, under salt stress, the OE seeds presented a significantly higher germination rate than did the WT seeds. On the stress media that included 200 mM NaCl, the germination rate of the OE seeds reached 60% compared with 20% for WT seeds after treatment for 48 h. In addition, the root length of OE lines was obviously longer than that of the WT plants on 150 mM NaCl media (Fig. 4B). When the 3-week-old seedlings were watered with 250 mM NaCl for 10 days, the leaves of WT began to turn yellow, but few yellow leaves were observed on the OE seedlings. After treatment for 15 days, more wilted and chlorotic leaves were observed on the WT than in the OE lines. After treatment with salt for 20 days, the OE plants presented a significantly higher survival rate (approximately 90%) than did the WT plants (approximately 40%) (Fig. 5A). In addition, under salt stress, the PRO accumulation in the OE plants was obviously higher than that in the WT (Fig. 5B), while the levels of MDA were obviously lower in the OE lines than in the WT (Fig. 5C).

**Figure 6:**
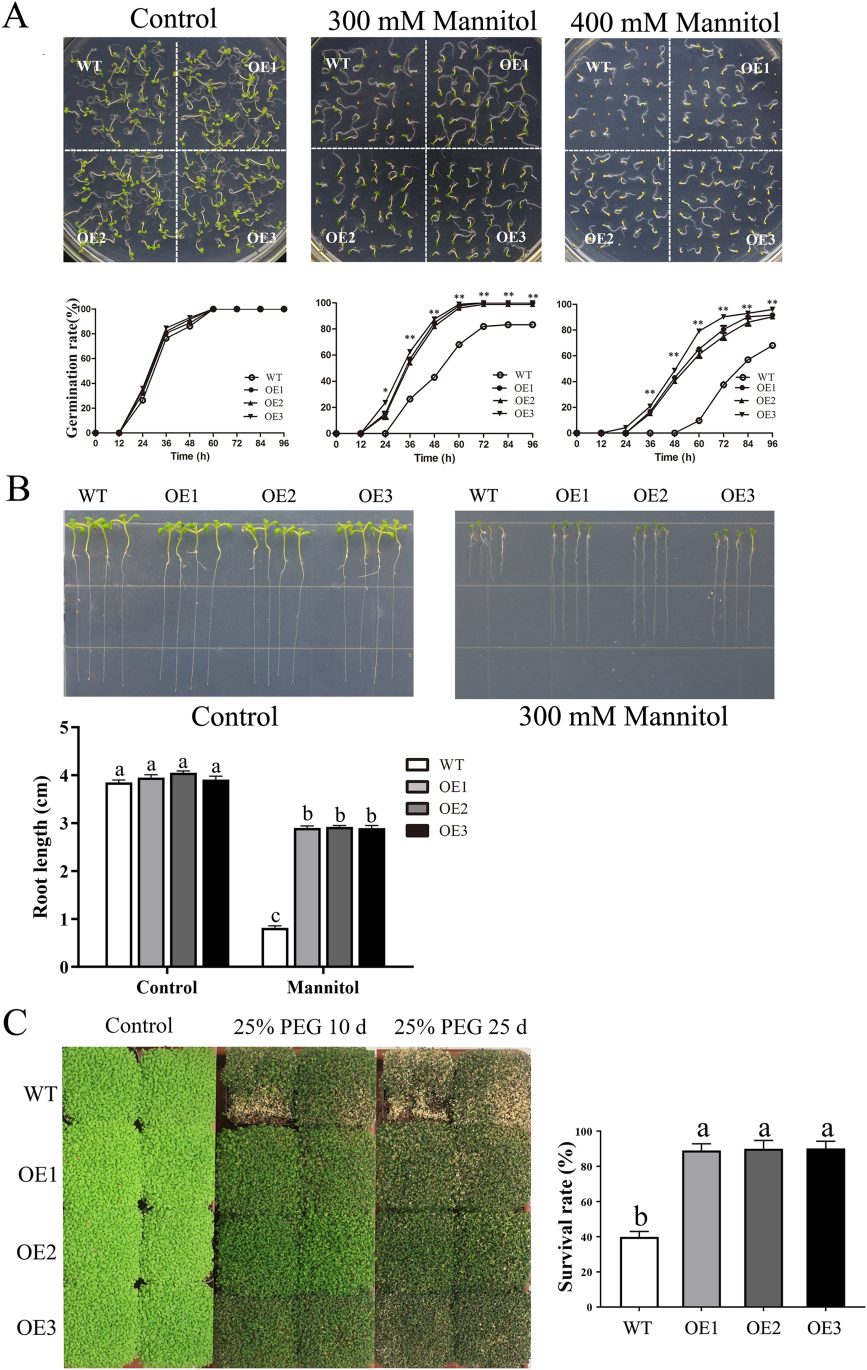
The phenotype of WT and *MdBBX1* transgenic plants in response to drought stress. (A) The seed germination of WT and the *MdBBX1* transgenic plants on 1/2 MS medium containing mannitol (0, 300, or 400 mM). Three independent experiments were conducted and each phenotype included 50 seeds. Vertical bars indicate the standard error of mean, ** and * indicate significant differences in comparison with WT at *P* < 0.01 and *P* < 0.05, respectively. (B) The root length of WT and the transgenic plants of *MdBBX1* in 1/2 MS medium containing 300 mM mannitol. Root growth of WT and the transgenic plants of *MdBBX1* was measured after 14 days. (C) The representative phenotypes of WT and OE seedlings were treated with 25% (w/v) PEG-6000 for 10 days and 25 days. All the columns were represented as mean values of three independent replicates and error bars indicate standard deviation. The letters above the columns represent significant differences (*P* < 0.05) based on Tukey’s multiple test.

Similarly, after mannitol treatment, the OE lines also displayed significantly higher germination rates and longer root lengths than did the WT line (Figs. 6A–6B). Moreover, the transgenic plants presented a higher survival rate than did the WT plants when treated with 25% PEG-6000 (Fig. 6C). When treated for 25 days, nearly 60% of WT plants wilted and died, while the leaves of OE plants yellowed slightly. The survival rate of the OE plants was approximately 90%, which was significantly higher than the survival rate of the WT plants. Taken together, these results indicated that overexpression of *MdBBX1* enhanced the abiotic stress resistance of the transgenic plants during germination and vegetative stage.

### Overexpression of *MdBBX1* in *Arabidopsis* decreased sensitivity to ABA

**Figure 7:**
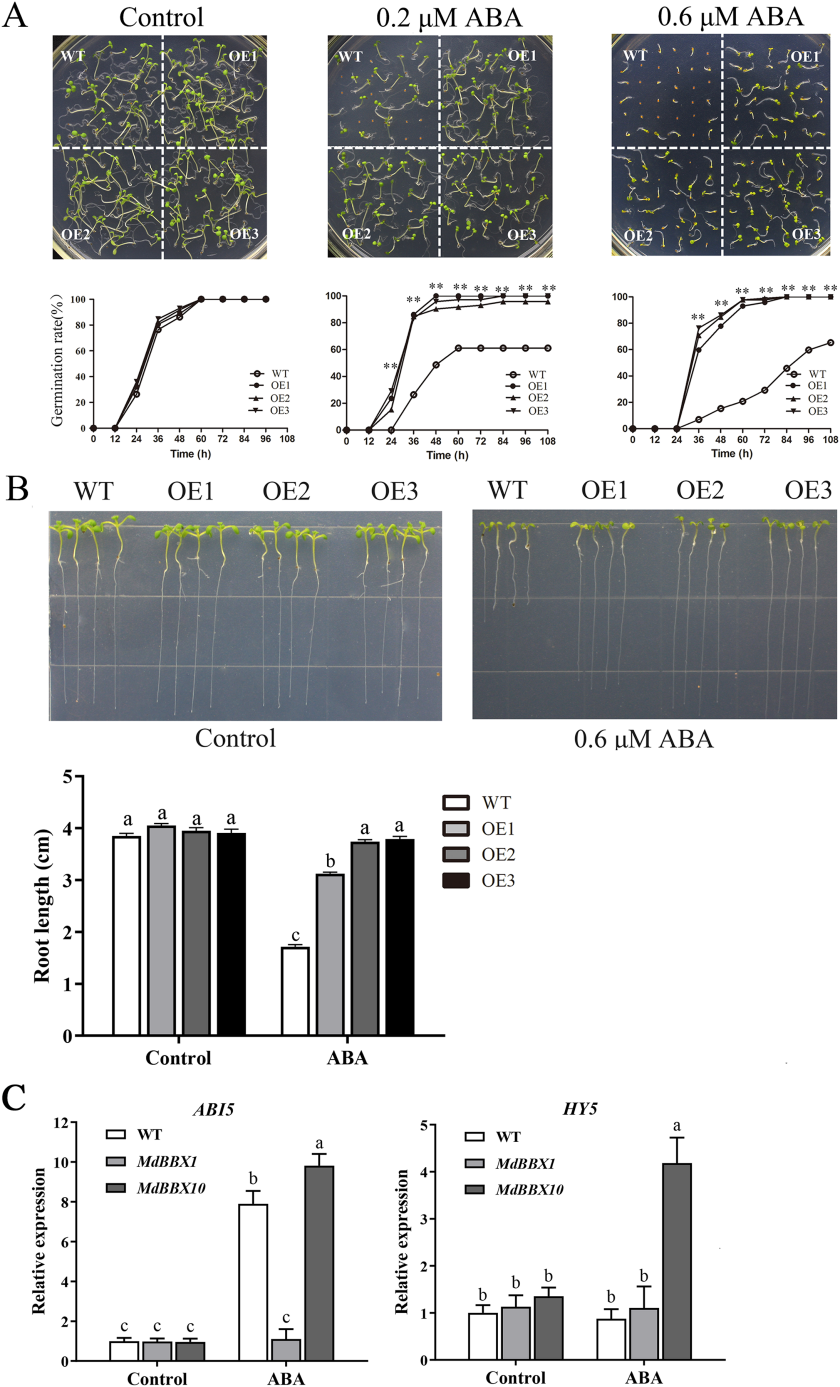
Germination phenotype of WT and transgenic plants in response to ABA. (A) Seed germination of WT and OE lines on 1/2 MS medium containing different ABA concentrations (0, 0.2 or 0.6 µM). Three independent experiments were conducted and each phenotype included 50 seeds. Vertical bars indicate the standard error of mean, ** indicate significant differences in comparison with WT at *P* < 0.01. (B) The root length of WT and OE lines on 1/2 MS medium containing 0.6 µM ABA. Root growth of WT and OE seedlings was measured after 14 days. (C) The transcriptional levels of *HY5* and *ABI5* in WT and OE lines after 100 µM ABA treatment for 3 h. Little difference was observed among different lines, so one of the OE *MdBBX1* transgenic lines was selected as the representative for gene expression analysis. The letters above the columns represent significant differences (*P* < 0.05) based on Tukey’s multiple test.

ABA plays a critical role in the physiological regulation of plant development in seed germination and in abiotic stress responses (Vishwakarma et al., 2017). To determine the potential function of *MdBBX1* in response to ABA, seeds of the OE and WT lines were plated on MS media either without or with ABA. Under MS media without ABA, the WT and OE lines displayed similar germination rates. However, under ABA treatment, the germination rates of the OE lines were significantly higher than those of the WT (Fig. 7A). Moreover, after the seedlings grew on MS media with ABA for 10 days, the primary root length of the OE seedlings was obviously greater than that of the WT seedlings (Fig. 7B). To determine the effect on root growth, the seeds of WT and OE were sown on MS media for 2 days first and then transferred to plates with media that included 0.6 μM ABA. The root length of the OE seedlings was still longer than that of the WT seedlings (Fig. S2). The above results suggested that overexpression of *MdBBX1* decreased ABA sensitivity in *Arabidopsis* during the germination and seedling stages.

In the ABA signaling pathway, HY5 and ABI5 are crucial for seed germination and seedling development (Chen & Xiong, 2008; Finkelstein, Gampala & Rock, 2002; Finkelstein & Lynch, 2000b). To determine whether the development of *MdBBX1*-overexpressing seedlings under abiotic stresses was related to HY5 or ABI5, the transcript levels of *ABI5* and *HY5* were measured after ABA treatment. The results revealed that the expression of *ABI5* was markedly reduced in the OE plants compared with the WT plants, while *HY5* changed only slightly (Fig. 7C). Another OE line overexpressing a different *MdBBX* family member (*MdBBX10*), which is ABA sensitive, was also evaluated under the same treatment (Liu et al., 2018). As shown in Fig. 7C, the changes in *ABI5* expression levels were opposite between *MdBBX1* and *MdBBX10*, which was reasonably expected in terms of a response to ABA treatment.

### Overexpression of *MdBBX1* reduced ROS accumulation in transgenic plants

**Figure 8:**
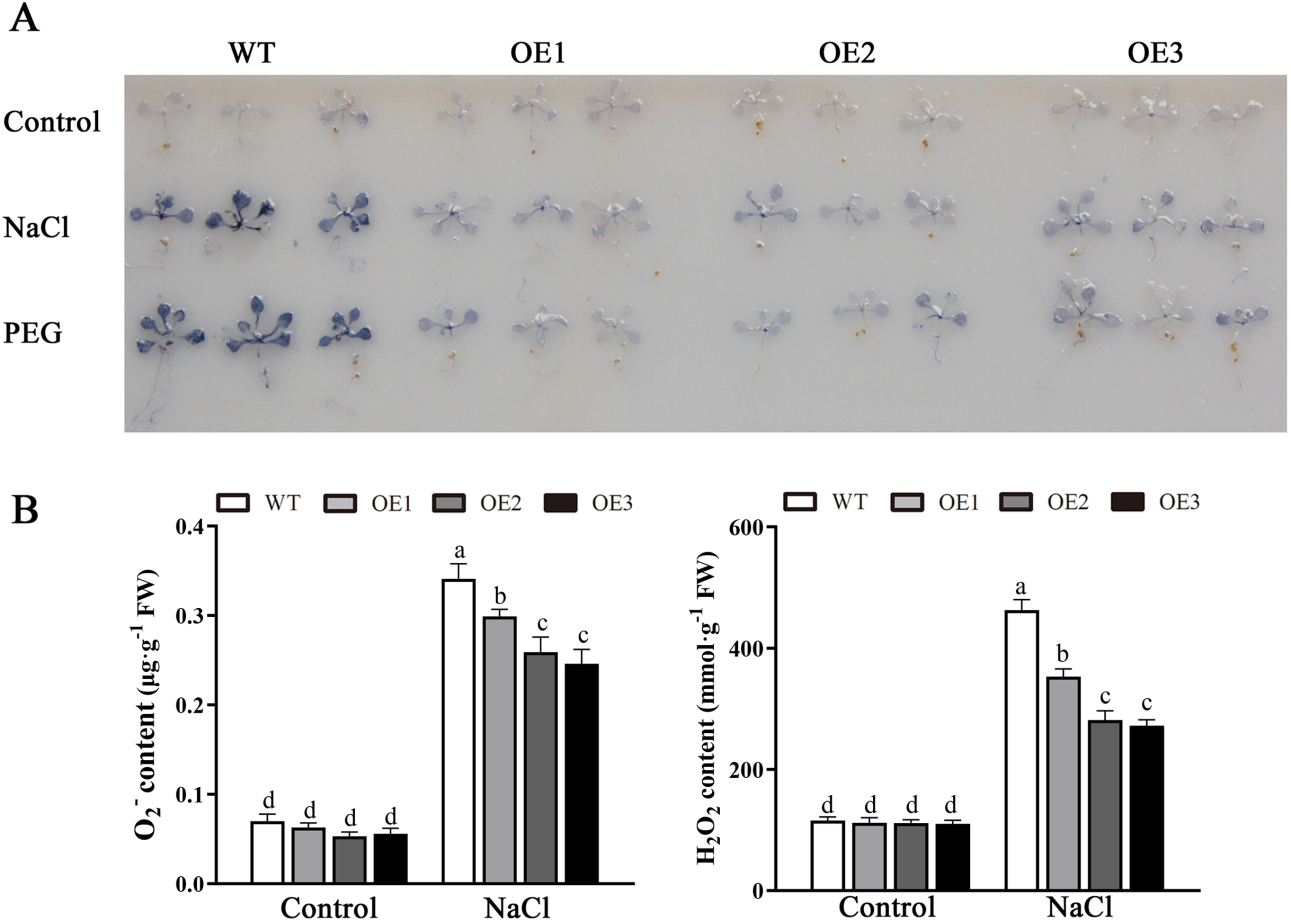
Analysis of ROS in WT and the *MdBBX1* transgenic plants after salt and drought stress. (A) NBT staining of O_2_^•−^ in WT and OE plants after the treatment with NaCl or PEG-6000 for 6 h. (B) The content of H_2_O_2_ and O_2_^•−^ in WT and transgenic lines after NaCl treatment. Each column represents the average of three replicates and error bars indicate standard deviation. The letters above the columns represent significant differences (*P* < 0.05) based on Tukey’s multiple test.

**Figure 9:**
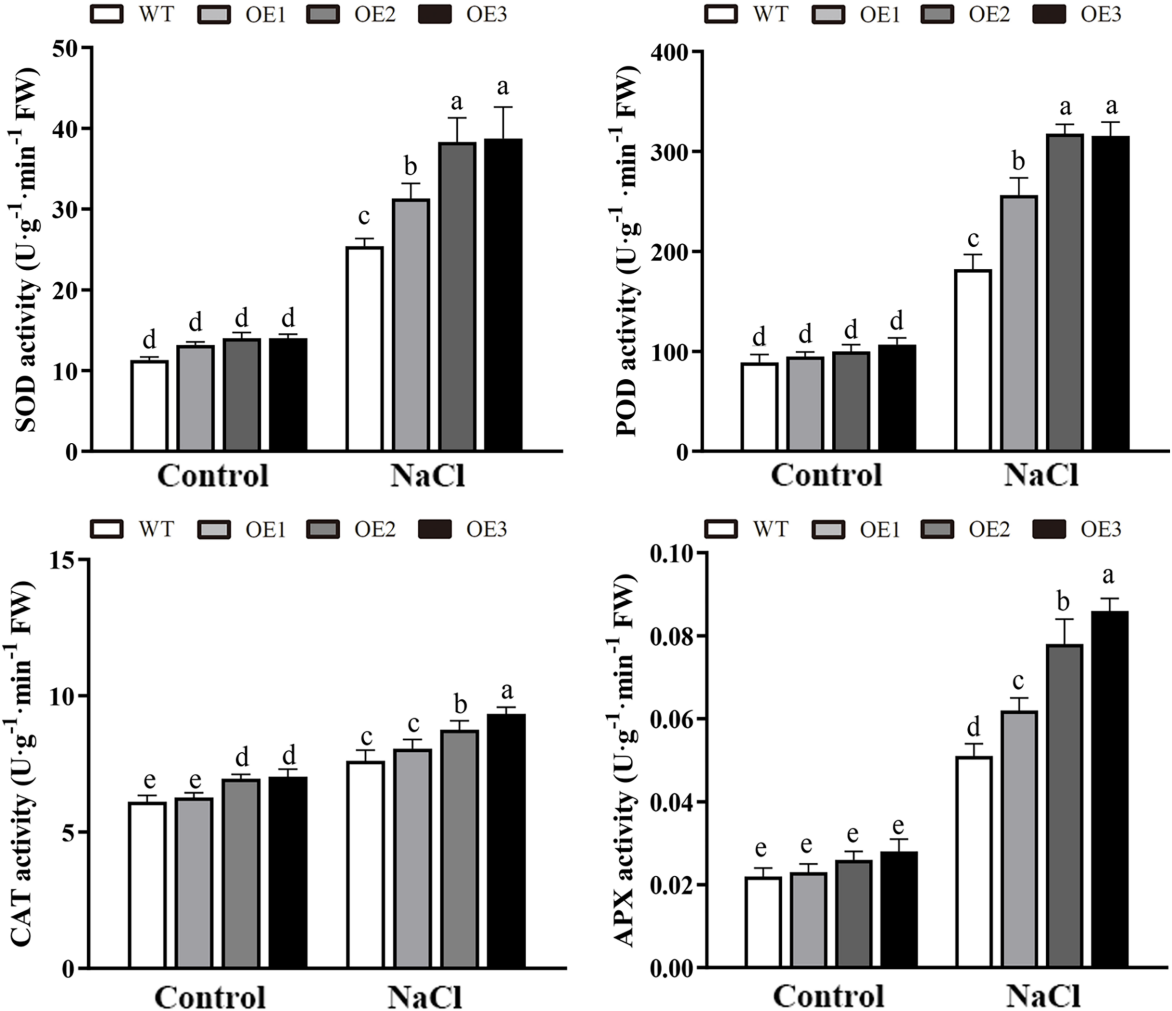
The activities of ROS-scavenging enzymes in WT and the *MdBBX1* transgenic plants after NaCl treatment. Each column represents the average of three replicates and error bars indicate standard deviation. The letters above the columns represent significant differences (*P* < 0.05) based on Tukey’s multiple test.

Various abiotic stresses often lead to the accumulation of excessive amounts of ROS, particularly H_2_O_2_ and O_2_^•−^, which has an important impact on plant growth and development (Mittler et al., 2004). To analyze whether *MdBBX1* responds to abiotic stress through the regulation of ROS levels, the accumulation of O_2_^•−^ in WT and OE plants was assessed *via* nitro blue tetrazolium (NBT) staining. No obvious difference was found between the WT and OE lines. However, under NaCl and PEG conditions, the OE lines accumulated lower levels of O_2_^•−^ than the WT did (Fig. 8A). Furthermore, the contents of H_2_O_2_ and O_2_^•−^ were measured, and the results showed that their contents in the OE lines were significantly lower than those in the WT (Figs. 8B, 8C). Similarly, under normal conditions, the activities of SOD, POD and APX were not notably different between the OE and WT lines. However, after salt treatment, the activities of SOD, POD and APX in the OE lines significantly increased compared with those in the WT lines (Fig. 9). Together, these results suggested that overexpression of *MdBBX1* could decrease the accumulation of ROS in transgenic plants by mediating the activities of ROS-scavenging enzymes.

## Discussion

Many BBXs are involved in the response to abiotic stresses in plants. For example, *AtBBX5* and *AtBBX24* are positive regulators that modulate the drought and salt stress resistance in *Arabidopsis* (Min et al., 2015; Nagaoka & Takano, 2003). Overexpression of *OsBBX25* in *Arabidopsis* increased the tolerance to abiotic stresses (Liu et al., 2012). Similarly, heterologous, constitutive expression of *CmBBX22* in *Arabidopsis* reduced seed germination and seedling growth under exogenous ABA, but improved plant drought tolerance (Liu et al., 2019b). A recent study found that overexpression of *MdBBX10* in *Arabidopsis* can promote the salt and drought tolerance, and the transgenic seedlings were shown to hypersensitive to exogenous ABA (Liu et al., 2019a). In this investigation, overexpression of *MdBBX1* also enhanced tolerance to abiotic stresses. However, the phenotype of *MdBBX1* overexpression plants was different from that of *MdBBX10* transgenic plants under exogenous ABA treatment. *MdBBX10* overexpressing plants were hypersensitive to exogenous ABA, while the *MdBBX1* transgenic plants were insensitive to ABA.

As a pivotal phytohormone, ABA is extensively involved in the regulation of plant growth and development (Wang et al., 2019), especially in response to various abiotic stresses and seed germination (Finkelstein, Gampala & Rock, 2002). During the initial stages of germination, the endogenous ABA content in seeds decreases rapidly and markedly after imbibition (Ali-Rachedi et al., 2004; Gubler, Millar & Jacobsen, 2005; Jacobsen et al., 2002). When exogenous ABA is added, seed germination and seedling growth can be repressed (Finkelstein, Gampala & Rock, 2002; Finkelstein & Lynch, 2000a). ABA-insensitive genes (*ABIs*), especially *ABI5*, play a vital role in ABA signaling and photomorphogenesis. *ABI5* is mainly expressed in dry seeds, and is involved in ABA-dependent growth arrest when seed dormancy is broken (Finkelstein, Gampala & Rock, 2002; Finkelstein & Lynch, 2000b). The efficiency of the ABA-dependent growth arrest is directly dependent on *ABI5* levels (Brocard, Lynch & Finkelstein, 2002; Lopez-Molina, Mongrand & Chua, 2001). *ABI5* markedly decreases after germination but can be induced by exogenous ABA (Finkelstein & Lynch, 2000b; Lopez-Molina, Mongrand & Chua, 2001). Moreover, the expression of *ABI5* can be activated by *HY5* through direct binding to its promoter in *Arabidopsis* (Chen et al., 2008). BBX family members also regulate the expression of *ABI5*. For example, *BBX19* from *Arabidopsis* suppresses seed germination by inducing expression of *ABI5* (Bai et al., 2019). However, in this investigation, the transcript level of *ABI5* significantly decreased in the *MdBBX1* OE plants compared with the WT plants, whereas it was significantly increased in the *MdBBX10* overexpression plants (Fig. 7C). Moreover, the transcript levels of *HY5* did not obviously change in the *MdBBX1* OE seedlings compared with the WT seedlings, but they were obliviously lower than those in the *MdBBX10* OE seedlings. These results are consistent with the phenotypes during seed germination after ABA treatment. In addition, the results of multiple sequence alignment revealed 17%, 36%, and 16% homology between HY5 and BBX1, BBX5, and BBX21, respectively (Fig. S3), which suggested that overexpression of *MdBBX1* may have little effect on the expression of endogenous genes. Taken together, these results suggested that *MdBBX1* may interfere with the expression of *ABI5* and *HY5* to promote seed germination and seeding growth in transgenic plants.

Abiotic stress can disrupt the normal homeostasis of plants, leading to the production of ROS, mainly comprises of H_2_O_2_ and O_2_^•−^ (Miller et al., 2010). Low concentrations of ROS act as critical signaling molecules that are beneficial to plant growth and development, especially when plants are exposed to extreme environmental conditions (Schippers et al., 2012). However, the accumulation of excessive amounts of ROS leads to very serious oxidative damage to plant cells and represses the normal growth of plants (Mullineaux & Baker, 2010). To alleviate oxidative damage, plant cells immediately employ a series of response mechanisms to suppress ROS production, such as the activation of ROS-scavenging enzymes (SOD, POD, CAT and APX) (Miller et al., 2010). Abiotic stress often increases the accumulation of ROS, thus causing membrane damage with lipid peroxidation, generating MDA. Our results suggested that the ROS and MDA contents were no significant different between the OE and WT lines under normal conditions. However, under salt treatment, compared with the WT line, the *MdBBX1* transgenic lines displayed a greater ROS-scavenging ability and antioxidant enzyme activities (Fig. 9), and a lower ROS accumulation (Fig. 8) and MDA levels (Fig. 5). Regulation of antioxidant capacity through improving the ROS-scavenging system might be a common mechanism to increase salt tolerance. Similar to our study, a previous study reported that overexpression of *ThSOS3* from *Tamarix hispida* improved the salt tolerance of transgenic plants by alleviating the accumulation of ROS, decreasing the accumulation of MDA accumulation and increasing the activity of antioxidant enzymes (Liu et al., 2020). In addition, overexpression of apple *MdMIPS1* also enhanced salt tolerance by increasing the activities of SOD, POD, and decreasing ROS and MDA contents in transgenic apple under salt stress (Hu et al., 2020). Taken together, these results indicated that *MdBBX1* provides salt stress resistance by enhancing ROS-scavenging system and alleviating oxidative stress.

## Conclusions

The transcript level of *MdBBX1* increased in response to various stresses. Overexpressing *MdBBX1* in *Arabidopsis* improved abiotic stress tolerance by regulating ABA signaling and the production of ROS. However, the detailed molecular mechanisms underlying these phenomena still need to be tested in future experiments.

## Supplemental Information

10.7717/peerj.12852/supp-1 Supplemental Information 1 Supplementary Table and Figures.

10.7717/peerj.12852/supp-2 Supplemental Information 2 Identification of the transgenic plants of *MdBBX1*. Phenotype of OE_1_, OE_2_ and OE_3_ plants; B. PCR products of transgenic plants; C. The expression of *MdBBX1* in the leaves of WT and transgenic plants.

10.7717/peerj.12852/supp-3 Supplemental Information 3 Roots length of WT and OE seedlings in medium containing ABA. The wild type and OE seedlings were grown on MS for 2 days and then were transferred to 1/2 MS medium containing 0.6 µm ABA.

10.7717/peerj.12852/supp-4 Supplemental Information 4 The homology among MdBBX1, AtBBX1, AtBBX5 and AtBBX21. Only 17%, 36%, 16% homology between MdBBX1 and AtBBX1, AtBBX5, and AtBBX21, respectively.

## Additional Information and Declarations

### Data Availability

The following information was supplied regarding data availability: The data is available at figshare: zhou, zhou (2022): MdBBX1 raw data. figshare. Dataset. https://doi.org/10.6084/m9.figshare.16575233.v1.

## References

[ref-1] Aguilar Díaz de León J, Borges CR (2020). Evaluation of oxidative stress in biological samples using the thiobarbituric acid reactive substances assay. Journal of Visualized Experiments.

[ref-2] Ali-Rachedi S, Bouinot D, Wagner MH, Bonnet M, Sotta B, Grappin P, Jullien M (2004). Changes in endogenous abscisic acid levels during dormancy release and maintenance of mature seeds: studies with the Cape Verde Islands ecotype, the dormant model of *Arabidopsis thaliana*. Planta.

[ref-59] An JP, Wang XF, Zhang XW, Bi SQ, You CX, Hao YJ (2019). MdBBX22 regulates UV-B-induced anthocyanin biosynthesis through regulating the function of MdHY5 and is targeted by MdBT2 for 26S proteasome-mediated degradation. Plant Biotechnology Journal.

[ref-3] An JP, Wang XF, Espley RV, Lin-Wang K, Bi SQ, You CX, Hao YJ (2020). An apple B-Box protein MdBBX37 modulates anthocyanin biosynthesis and hypocotyl elongation synergistically with *MdMYBs* and *MdHY5*. Plant and Cell Physiology.

[ref-4] Bai M, Sun J, Liu J, Ren H, Wang K, Wang Y, Wang C, Dehesh K (2019). The B-box protein BBX19 suppresses seed germination via induction of *ABI5*. Plant Journal.

[ref-5] Bai Y, Xiao S, Zhang Z, Zhang Y, Sun H, Zhang K, Wang X, Bai Z, Li C, Liu L (2020). Melatonin improves the germination rate of cotton seeds under drought stress by opening pores in the seed coat. PeerJ.

[ref-6] Brocard IM, Lynch TJ, Finkelstein RR (2002). Regulation and role of the *Arabidopsis* abscisic acid-insensitive 5 gene in abscisic acid, sugar, and stress response. Plant Physiology.

[ref-7] Chang CS, Li YH, Chen LT, Chen WC, Hsieh WP, Shin J, Jane WN, Chou SJ, Choi G, Hu JM, Somerville S, Wu SH (2008). LZF1, a HY5-regulated transcriptional factor, functions in *Arabidopsis* de-etiolation. Plant Journal.

[ref-8] Chen H, Xiong L (2008). Role of HY5 in abscisic acid response in seeds and seedlings. Plant Signaling & Behavior.

[ref-9] Chen H, Zhang J, Neff MM, Hong SW, Zhang H, Deng XW, Xiong L (2008). Integration of light and abscisic acid signaling during seed germination and early seedling development. Proceedings of the National Academy of Sciences of the United States of America.

[ref-10] Chen J, Chen JY, Wang JN, Kuang JF, Shan W, Lu WJ (2012). Molecular characterization and expression profiles of MaCOL1, a CONSTANS-like gene in banana fruit. Gene.

[ref-11] Chu Z, Wang X, Li Y, Yu H, Li J, Lu Y, Li H, Ouyang B (2016). Genomic organization, phylogenetic and expression analysis of the B-BOX gene family in tomato. Frontiers in Plant Science.

[ref-12] Crocco CD, Botto JF (2013). BBX proteins in green plants: Insights into their evolution, structure, feature and functional diversification. Gene.

[ref-13] Crocco CD, Holm M, Yanovsky MJ, Botto JF (2011). Function of B-BOX under shade. Plant Signaling & Behavior.

[ref-14] Crocco CD, Locascio A, Escudero CM, Alabadi D, Blazquez MA, Botto JF (2015). The transcriptional regulator BBX24 impairs DELLA activity to promote shade avoidance in Arabidopsis thaliana. Nature Communications.

[ref-15] Crocco CD, Ocampo GG, Ploschuk EL, Mantese A, Botto JF (2018). Heterologous Expression of AtBBX21 enhances the rate of photosynthesis and alleviates photoinhibition in *Solanum tuberosum*. Plant Physiology.

[ref-16] Datta S, Hettiarachchi C, Johansson H, Holm M (2007). SALT TOLERANCE HOMOLOG2, a B-box protein in *Arabidopsis* that activates transcription and positively regulates light-mediated development. Plant Cell.

[ref-17] Datta S, Johansson H, Hettiarachchi C, Irigoyen ML, Desai M, Rubio V, Holm M (2008). LZF1/SALT TOLERANCE HOMOLOG3, an *Arabidopsis* B-box protein involved in light-dependent development and gene expression, undergoes COP1-mediated ubiquitination. Plant Cell.

[ref-61] Deepak S, Kottapalli K, Rakwal R, Oros G, Rangappa K (2007). Real-Time PCR: revolutionizing detection and expression analysis of genes. Current Genomics.

[ref-18] Du F, Xu JN, Zhan CY, Yu ZB, Wang XY (2014). An obesity-like gene *MdTLP7* from apple (*Malus* x *domestica*) enhances abiotic stress tolerance. Biochemical and Biophysical Research Communications.

[ref-19] Fang H, Dong Y, Yue X, Hu J, Jiang S, Xu H, Wang Y, Su M, Zhang J, Zhang Z, Wang N, Chen X (2019). The B-box zinc finger protein MdBBX20 integrates anthocyanin accumulation in response to ultraviolet radiation and low temperature. Plant Cell and Environment.

[ref-20] Finkelstein RR, Gampala SS, Rock CD (2002). Abscisic acid signaling in seeds and seedlings. Plant Cell.

[ref-21] Finkelstein RR, Lynch TJ (2000a). Abscisic acid inhibition of radicle emergence but not seedling growth is suppressed by sugars. Plant Physiology.

[ref-22] Finkelstein RR, Lynch TJ (2000b). The *Arabidopsis* abscisic acid response gene *ABI5* encodes a basic leucine zipper transcription factor. Plant Cell.

[ref-23] Gangappa SN, Botto JF (2014). The BBX family of plant transcription factors. Trends in Plant Science.

[ref-24] Gangappa SN, Crocco CD, Johansson H, Datta S, Hettiarachchi C, Holm M, Botto JF (2013). The *Arabidopsis* B-BOX protein BBX25 interacts with HY5, negatively regulating BBX22 expression to suppress seedling photomorphogenesis. Plant Cell.

[ref-25] Gasic K, Hernandez A, Korban SSJPMBR (2004). RNA extraction from different apple tissues rich in polyphenols and polysaccharides for cDNA library construction. Plant Molecular Biology Reporter.

[ref-26] Gubler F, Millar AA, Jacobsen JV (2005). Dormancy release, ABA and pre-harvest sprouting. Current Opinion in Plant Biology.

[ref-27] Heng Y, Jiang Y, Zhao X, Zhou H, Wang X, Deng XW, Xu D (2019a). BBX4, a phyB-interacting and modulated regulator, directly interacts with PIF3 to fine tune red light-mediated photomorphogenesis. Proceedings of the National Academy of Sciences of the United States of America.

[ref-28] Heng Y, Lin F, Jiang Y, Ding M, Yan T, Lan H, Zhou H, Zhao X, Xu D, Deng XW (2019b). B-Box containing proteins BBX30 and BBX31, acting downstream of HY5, negatively regulate photomorphogenesis in Arabidopsis. Plant Physiology.

[ref-29] Hodges DM, Delong JM, Prange F (1999). Improving the thiobarbituric acid-reactive-substances assay for estimating lipid peroxidation in plant tissues containing anthocyanin and other interfering compounds. Planta.

[ref-30] Hu L, Zhou K, Liu Y, Yang S, Zhang J, Gong X, Ma F (2020). Overexpression of *MdMIPS1* enhances salt tolerance by improving osmosis, ion balance, and antioxidant activity in transgenic apple. Plant Science.

[ref-31] Jacobsen JV, Pearce DW, Poole AT, Pharis RP, Mander LN (2002). Abscisic acid, phaseic acid and gibberellin contents associated with dormancy and germination in barley. Physiologia Plantarum.

[ref-32] Job N, Yadukrishnan P, Bursch K, Datta S, Johansson H (2018). Two B-Box proteins regulate photomorphogenesis by oppositely modulating HY5 through their diverse C-terminal domains. Plant Physiology.

[ref-33] Khanna R, Kronmiller B, Maszle DR, Coupland G, Holm M, Mizuno T, Wu SH (2009). The Arabidopsis B-box zinc finger family. Plant Cell.

[ref-34] Li PT, Rashid MHO, Chen TT, Lu QW, Ge Q, Gong WK, Liu AY, Gong JW, Shang HH, Deng XY, Li JW, Li SQ, Xiao XH, Liu RX, Zhang Q, Duan L, Zou XY, Zhang Z, Jiang X, Zhang Y, Peng RH, Shi YZ, Yuan YL (2019). Transcriptomic and biochemical analysis of upland cotton (*Gossypium hirsutum*) and a chromosome segment substitution line from *G. hirsutum* x *G. barbadense* in response to *Verticillium dahliae* infection. BMC Plant Biology.

[ref-35] Liu X, Li R, Dai Y, Chen X, Wang X (2018). Genome-wide identification and expression analysis of the B-box gene family in the Apple (*Malus domestica Borkh*.) genome. Molecular Genetics and Genomics.

[ref-36] Liu X, Li R, Dai Y, Yuan L, Sun Q, Zhang S, Wang X (2019a). A B-box zinc finger protein, MdBBX10, enhanced salt and drought stresses tolerance in *Arabidopsis*. Plant Molecular Biology.

[ref-62] Liu Y, Chen H, Ping Q, Zhang Z, Guan Z, Fang W, Chen S, Chen F, Jiang J, Zhang F (2019b). The heterologous expression of CmBBX22 delays leaf senescence and improves drought tolerance in Arabidopsis. Plant Cell Reports.

[ref-37] Liu Y, Xing L, Li J, Dai S (2012). Rice B-box zinc finger protein OsBBX25 is involved in the abiotic response. Zhiwu Xuebao.

[ref-38] Liu Z, Xie Q, Tang F, Wu J, Dong W, Wang C, Gao C (2020). The *ThSOS3* Gene improves the salt tolerance of transgenic *Tamarix hispida* and *Arabidopsis thaliana*. Frontiers of Plant Science.

[ref-39] Livak KJ, Schmittgen TD (2001). Analysis of relative gene expression data using real-time quantitative PCR and the 2−ΔΔCT Method. Methods.

[ref-40] Lopez-Molina L, Mongrand S, Chua NH (2001). A postgermination developmental arrest checkpoint is mediated by abscisic acid and requires the ABI5 transcription factor in *Arabidopsis*. Proceedings of the National Academy of Sciences of the United States of America.

[ref-41] Ma Y, Wang P, Wang M, Sun M, Gu Z, Yang R (2019). GABA mediates phenolic compounds accumulation and the antioxidant system enhancement in germinated hulless barley under NaCl stress. Food Chemistry.

[ref-42] Miller G, Suzuki N, Ciftci-Yilmaz S, Mittler R (2010). Reactive oxygen species homeostasis and signalling during drought and salinity stresses. Plant Cell and Environment.

[ref-43] Min JH, Chung JS, Lee KH, Kim CS (2015). The CONSTANS-like 4 transcription factor, AtCOL4, positively regulates abiotic stress tolerance through an abscisic acid-dependent manner in Arabidopsis. Journal of Integrative Plant Biology.

[ref-44] Mittler R, Vanderauwera S, Gollery M, Van Breusegem F (2004). Reactive oxygen gene network of plants. Trends in Plant Science.

[ref-45] Mullineaux PM, Baker NR (2010). Oxidative stress: antagonistic signaling for acclimation or cell death?. Plant Physiology.

[ref-46] Nagaoka S, Takano T (2003). Salt tolerance-related protein STO binds to a *Myb* transcription factor homologue and confers salt tolerance in *Arabidopsis*. Journal of Experimental Botany.

[ref-47] Sarmiento F (2013). The BBX subfamily IV: additional cogs and sprockets to fine-tune light-dependent development. Plant Signaling & Behavior.

[ref-48] Schippers JH, Nguyen HM, Lu D, Schmidt R, Mueller-Roeber B (2012). ROS homeostasis during development: an evolutionary conserved strategy. Cellular and Molecular Life Sciences.

[ref-49] Shalmani A, Fan S, Jia P, Li G, Muhammad I, Li Y, Sharif R, Dong F, Zuo X, Li K, Chen KM, Han M (2018). Genome identification of B-BOX gene family members in seven Rosaceae species and their expression qnalysis in response to flower induction in *Malus domestica*. Molecules.

[ref-50] Vishwakarma K, Upadhyay N, Kumar N, Yadav G, Singh J, Mishra RK, Kumar V, Verma R, Upadhyay RG, Pandey M, Sharma S (2017). Abscisic acid signaling and abiotic stress tolerance in plants: a review on current knowledge and future prospects. Frontiers in Plant Science.

[ref-51] Wang C, He X, Li Y, Wang L, Guo X, Guo X (2018). The cotton MAPK kinase GhMPK20 negatively regulates resistance to *Fusarium oxysporum* by mediating the *MKK4-MPK20-WRKY40* cascade. Molecular Plant Pathology.

[ref-52] Wang H, Zhang Z, Li H, Zhao X, Liu X, Ortiz M, Lin C, Liu B (2013). CONSTANS-LIKE 7 regulates branching and shade avoidance response in *Arabidopsis*. Journal of Experimental Botany.

[ref-53] Wang Y, Yang L, Chen X, Ye T, Zhong B, Liu R, Wu Y, Chan Z (2016). Major latex protein-like protein 43 (MLP43) functions as a positive regulator during abscisic acid responses and confers drought tolerance in *Arabidopsis thaliana*. Journal of Experimental Botany.

[ref-54] Wang YY, Xiong F, Ren QP, Wang XL (2019). Regulation of flowering transition by alternative splicing: the role of the U2 auxiliary factor. Journal of Experimental Botany.

[ref-55] Wei CQ, Chien CW, Ai LF, Zhao J, Zhang Z, Li KH, Burlingame AL, Sun Y, Wang ZY (2016). The *Arabidopsis* B-box protein BZS1/BBX20 interacts with HY5 and mediates strigolactone regulation of photomorphogenesis. Journal of Genetics and Genomics.

[ref-60] Xu D, Jiang Y, Li J, Lin F, Holm M, Deng XW (2016). BBX21, an Arabidopsis B-box protein, directly activates HY5 and is targeted by COP1 for 26S proteasome-mediated degradation. Proceedings of the National Academy of Sciences of the United States of America.

[ref-56] Yadav A, Bakshi S, Yadukrishnan P, Lingwan M, Dolde U, Wenkel S, Masakapalli SK, Datta S (2019). The B-Box-Containing microprotein miP1a/BBX31 regulates photomorphogenesis and UV-B protection. Plant Physiology.

[ref-57] Yuan H, Zhao K, Lei H, Shen X, Liu Y, Liao X, Li T (2013). Genome-wide analysis of the *GH3* family in apple (*Malus* x *domestica*). BMC Genomics.

[ref-58] Zhang X, Huai J, Shang F, Xu G, Tang W, Jing Y, Lin R (2017). A PIF1/PIF3-HY5-BBX23 transcription factor cascade affects photomorphogenesis. Plant Physiology.

