# Supplementary material for: B-box containing protein 1 from Malus domestica (MdBBX1) is involved in the abiotic stress response
Source: PeerJ. 2022 Feb 1;10:e12852. doi: 10.7717/peerj.12852 (PMC8815370; doi:10.7717/peerj.12852)

**Supplementary materials**

Table S1 Primers used in this study

| Primer name | Sequence (5′ to 3′) | Description |
| --- | --- | --- |
| *MdBBX1-*forward | GGGGTACCATGGCGTTGAAGCTTT | cDNA sequence primer |
| *MdBBX1-*reverse | GGAATTCAAACGACGGAACGATGC | cDNA sequence primer |
| *MdBBX1-GFP-*forward | GCTCTAGAATGGCGTTGAAGCTTTG | cDNA sequence primer |
| *MdBBX1-GFP-*reverse | GGGGTACCAAACGACGGAACG | cDNA sequence primer |
| q*MdBBX1-*forward | GAGACAGAGTGTTTCATC | qRT*-*PCR primer |
| q*MdBBX1-*reverse | TTGACTTGTTGTAAGGATT | qRT*-*PCR primer |
| q*MdActin* - forward | TGACCGAATGAGCAAGGAAATTACT | qRT*-*PCR primer |
| q*MdActin* - reverse | TACTCAGCTTTGGCAATCCACATC | qRT*-*PCR primer |
| q*AtGAPDH-*forward | TGGTTGATCTCGTTGTGCAGGTC | qRT*-*PCR primer |
| q*AtGAPDH-*reverse | GTCAGCCAAGTCAACAACTCT | qRT*-*PCR primer |
| q*ABI5*- forward | ATCCTCTATCTACTCATTG | qRT*-*PCR primer |
| q*ABI5*- reverse | CAGAGGAGAATAATAACAAT | qRT*-*PCR primer |
| qHY*5*- forward | ACAGAGTGAAAGACTTGGA | qRT*-*PCR primer |
| qHY*5*- reverse | TGTCTAAGCATCTGGTTCT | qRT*-*PCR primer |
| q*actin2/8*- forward | GGTAAACATTGTGCTCAGTGGTGG | qRT*-*PCR primer |
| qactin2/8- forward | AACGACCTTAATCTTCATGCTGC | qRT*-*PCR primer |

Figure. S1 Identification of the transgenic plants of *MdBBX1*. A. Phenotype of OE_1_, OE_2_ and OE_3_ plants; B. PCR products of transgenic plants; C. qRT-PCR analysis of the expression of *MdBBX1* in the leaves of WT and transgenic plants.


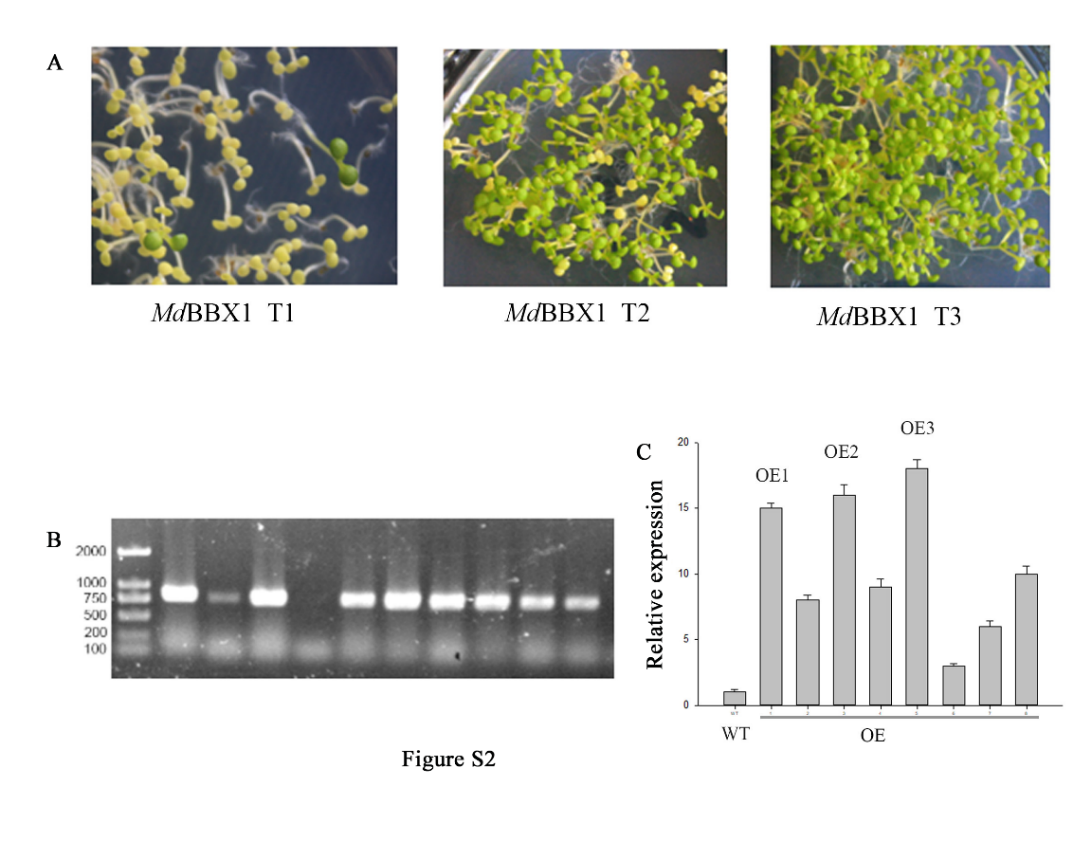


Figure. S2 Roots length of WT and OE seedlings in medium containing ABA. The wild type and OE seedlings were grown on MS for 2 days and then were transferred to 1/2 MS medium containing 0.6 µM ABA


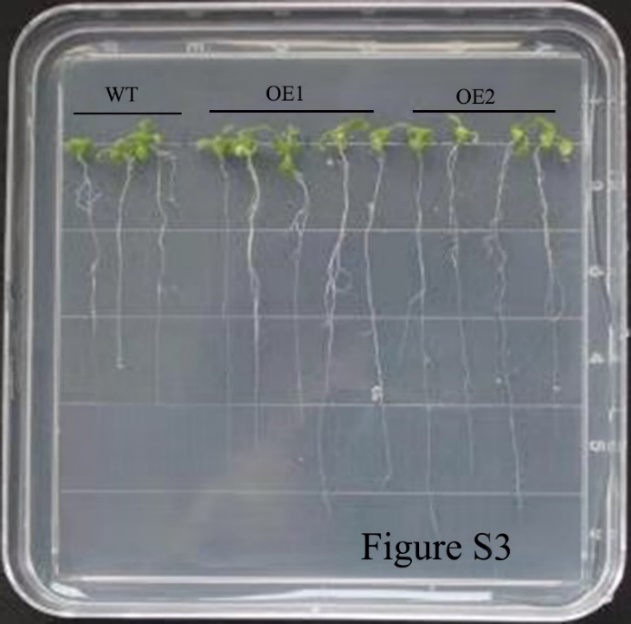


Fig. S3 The homology among MdBBX1, AtBBX1, AtBBX5 and AtBBX21. Only 17%, 36%, 16% homology among *Mt*BBX1, BBX1, BBX5, and BBX21 respectively.


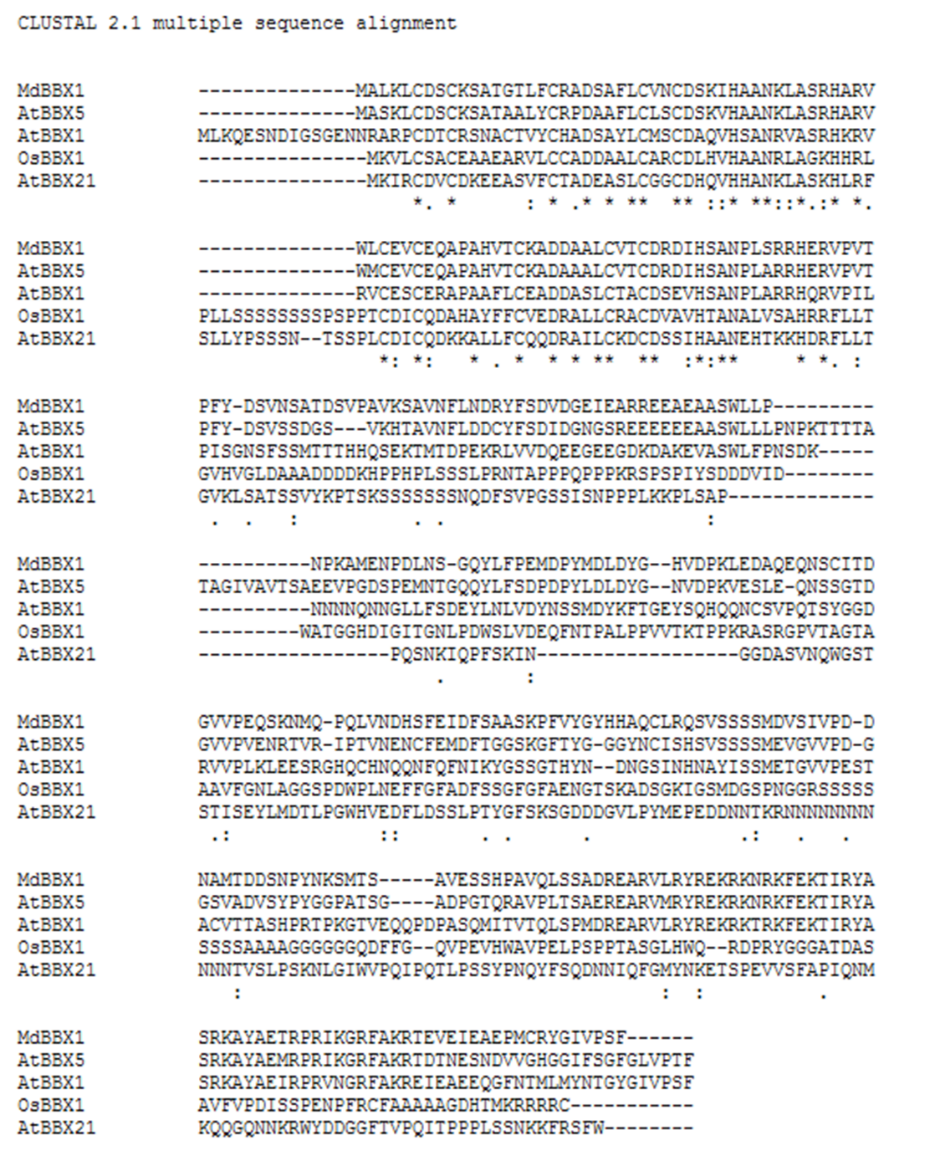

Supplement: Supplemental Information 1 [file peerj-10-12852-s001.docx]
