# Supplementary figures and images for: B-box containing protein 1 from Malus domestica (MdBBX1) is involved in the abiotic stress response
Source: PeerJ. 2022 Feb 1;10:e12852. doi: 10.7717/peerj.12852 (PMC8815370; doi:10.7717/peerj.12852)

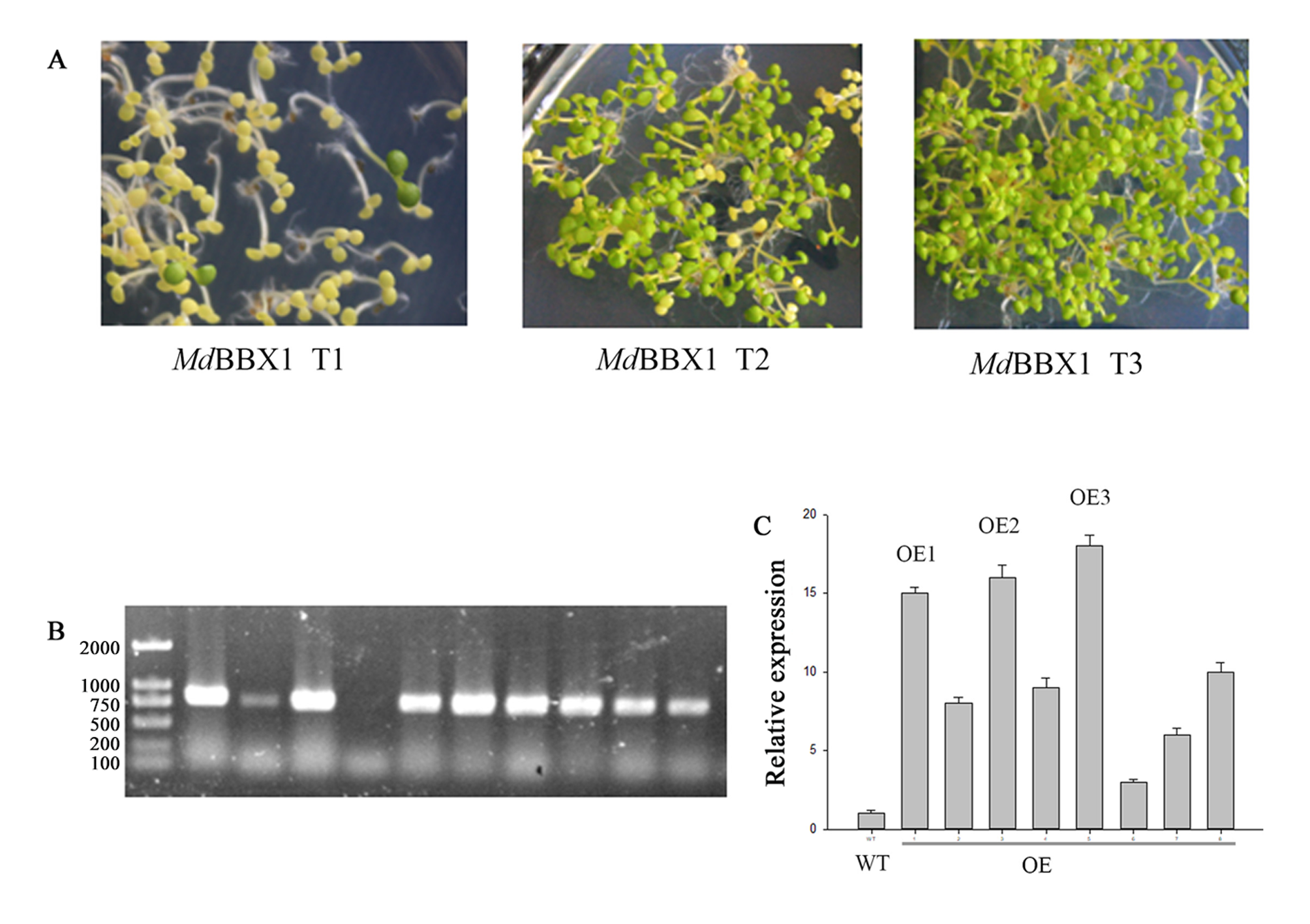

Supplement: Supplemental Information 2 — Phenotype of OE1, OE2 and OE3 plants; B. PCR products of transgenic plants; C. The expression of MdBBX1 in the leaves of WT and transgenic plants. [file peerj-10-12852-s002.png]

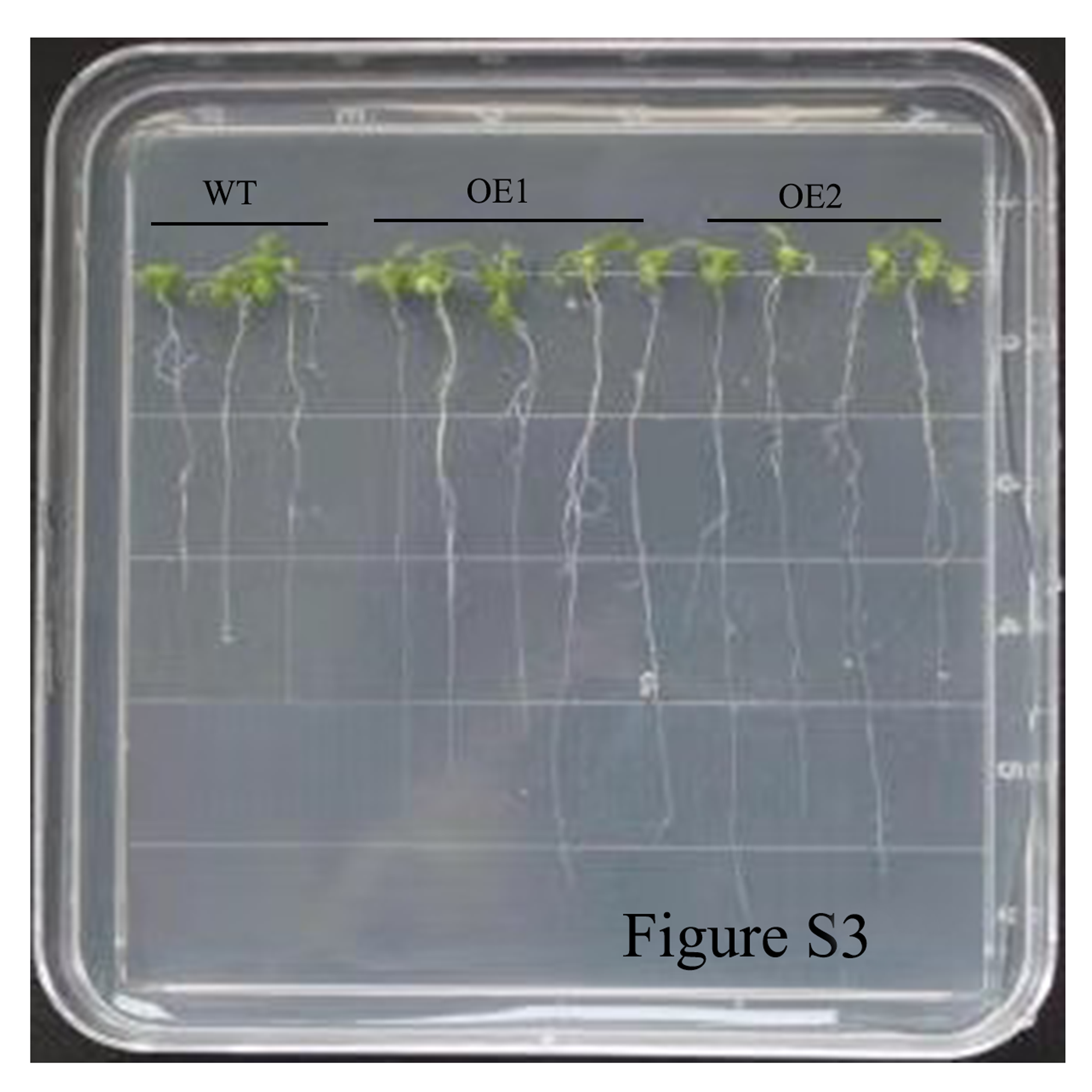

Supplement: Supplemental Information 3 — The wild type and OE seedlings were grown on MS for 2 days and then were transferred to 1/2 MS medium containing 0.6 µm ABA. [file peerj-10-12852-s003.png]

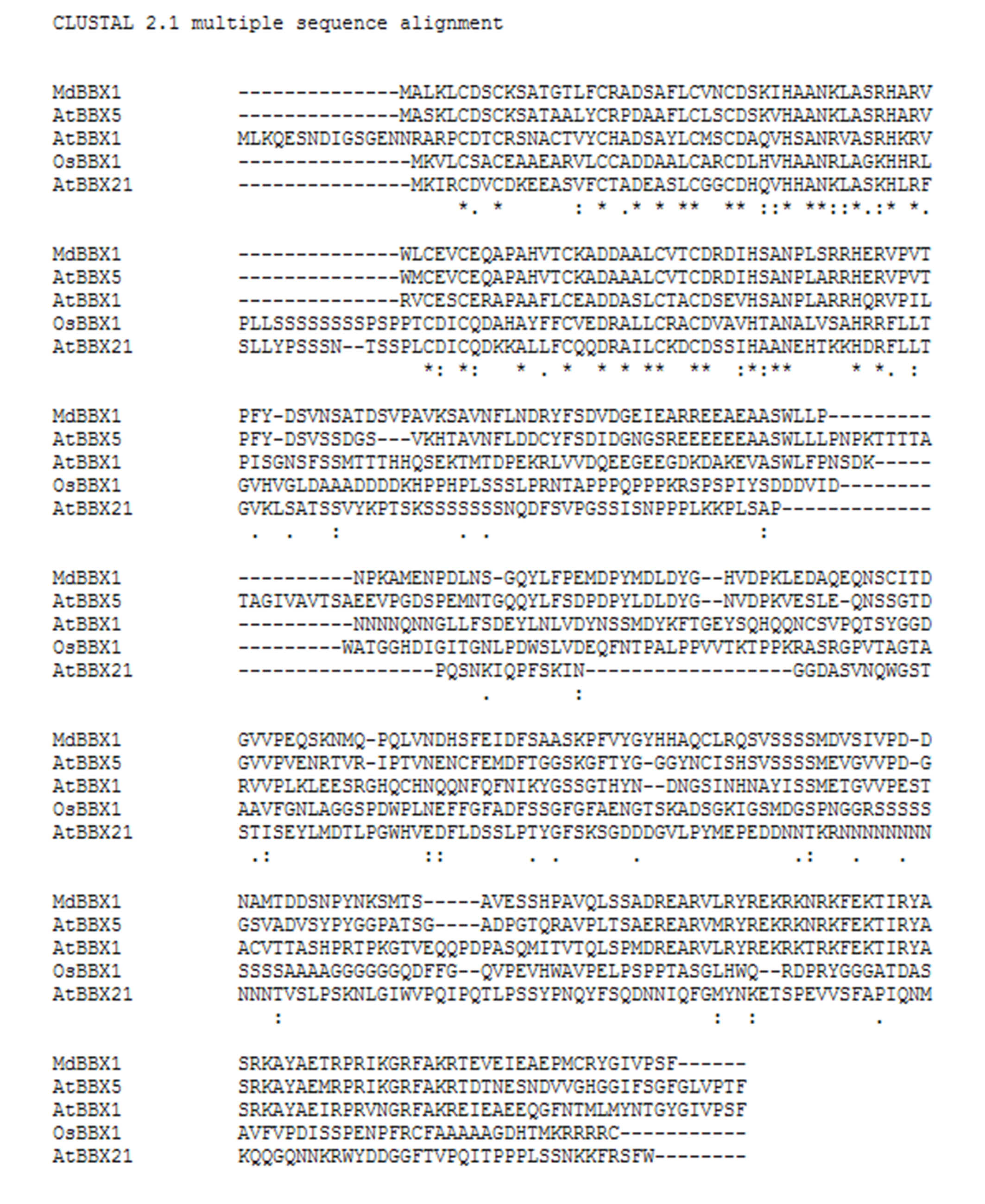

Supplement: Supplemental Information 4 — Only 17%, 36%, 16% homology between MdBBX1 and AtBBX1, AtBBX5, and AtBBX21, respectively. [file peerj-10-12852-s004.png]
